# Plasma‐Derived Exosomal i‐tRF‐LeuCAA as Biomarker for Glioma Diagnosis and Promoter of Epithelial‐Mesenchymal Transition via TPM4 Regulation

**DOI:** 10.1111/cns.70356

**Published:** 2025-04-09

**Authors:** Hongyu Liu, Wentao Hu, Lijun Zhang, Ze Li, Jialin Liu, Ling Chen

**Affiliations:** ^1^ Medical School of Chinese PLA Beijing China; ^2^ Department of Neurosurgery Hainan Hospital of Chinese PLA General Hospital Sanya Hainan China; ^3^ Department of Neurosurgery First Medical Center of Chinese PLA General Hospital Beijing China; ^4^ School of Medicine, Nankai University Tianjin China; ^5^ Laboratory of Oncology The First Medical Center of Chinese PLA General Hospital Beijing China

**Keywords:** bioinformatics analysis, biomarker, exosome, glioma, tsRNA

## Abstract

**Aims:**

This study aimed to discover plasma‐derived exosomal tsRNAs that serve as novel diagnostic biomarkers for glioma and to investigate the mechanism by which tsRNAs regulate glioma development.

**Methods:**

Differentially expressed tsRNAs in the plasma exosomes of glioma patients were identified using small RNA array sequencing. Bioinformatics analyses were used to predict the biological function of tsRNAs. The changes in the phenotypes of glioma cells treated with a tsRNA mimic and inhibitor were detected. The diagnostic and prognostic characteristics of potential target genes and their related functions in gliomas were further analyzed. The cell and animal experiments were used to analyze the molecular mechanisms.

**Results:**

Among the 453 differentially expressed tsRNAs identified in the plasma‐derived exosomes of glioma patients using small RNA sequencing, i‐tRF‐LeuCAA was associated with the prognosis and molecular diagnostic characteristics of glioma patients and promoted the migration, invasion, and proliferation of glioma cells and inhibited their apoptosis. In addition, TPM4 is a potential target of i‐tRF‐LeuCAA and is related to epithelial–mesenchymal transition in gliomas.

**Conclusions:**

i‐tRF‐LeuCAA could be served as a non‐invasive biomarker in the diagnosis and prognosis of glioma. i‐tRF‐LeuCAA may indirectly regulate TPM4 expression and influence epithelial–mesenchymal transition, which may promote glioma progression.

## Graphical abstract

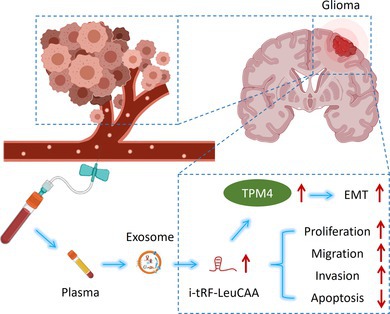

## 1 Introduction

The most common primary malignant brain tumor is glioma, and glioblastoma (GBM) is the most malignant glioma with the worst prognosis. Although surgery, chemotherapy, and radiotherapy can improve patient prognosis, the median overall survival (OS) is only 14.6 months [1]. The poor prognosis of glioma is due to the absence of early diagnostic methods and effective tools for sensitive efficacy monitoring, as well as a lack of understanding of the molecular mechanisms underlying its pathogenesis. Currently, there is a lack of specific biomarkers for diagnosing or treating gliomas. Therefore, it is important to identify biomarkers with clinical application value and investigate the molecular mechanisms underlying glioma development.

tRNA‐derived small RNA (tsRNA) is a newly discovered class of small non‐coding RNAs (sncRNAs) [2]. tsRNA plays a crucial role in tumors by participating in various biological functions, including gene expression regulation, epigenetic regulation, and translation inhibition [3]. In addition, tsRNAs can be enclosed in microcarriers, such as exosomes, or form stabilized complexes to protect them from degradation by RNA enzymes so that they can be secreted as a stable entity in the peripheral blood [4]. The heterogeneity and stability of plasma‐derived exosomal tsRNAs make them suitable candidates for tumor diagnostic biomarkers. However, their expression profile in the plasma‐derived exosomes of glioma patients remains unclear and requires further investigation.

Exosomes are small cell‐derived vesicles secreted into the extracellular environment [5]. Exosomes can cross the blood–brain barrier and regulate the initiation and progression of glioma by transporting bioactive molecules to different cell subsets [6, 7]. tsRNAs, which have a stable stem‐loop structure, can bind to nucleoproteins to form stabilized complexes and exist in the exosomes of body fluids, such as plasma, at high concentrations [8]. tsRNAs in salivary exosomes are reliable biomarkers in the clinical diagnosis and prognosis of patients with esophageal squamous cell carcinoma and predict the sensitivity to adjuvant chemoradiotherapy [9]. tsRNA expression levels in the plasma exosomes of patients with liver cancer are significantly increased [10], indicating that tsRNAs are novel cancer biomarkers. However, no relevant studies have shown that plasma‐derived exosomes can be used to diagnose gliomas.

tsRNAs can affect the progression of many types of cancer by regulating the proliferation, metastasis, apoptosis, and metabolism of cancer cells [11]. tsRNAs regulate various signaling pathways related to cell proliferation and migration, transcriptome regulation, and the cell cycle by binding target mRNA or proteins, affecting tumor initiation and development [11]. For example, low tRFdb‐3003a/b expression in gliomas promotes the proliferation of glioma cells by regulating the expression level of VAV2; however, more mechanisms need to be investigated [12].

This study aimed to discover plasma‐derived exosomal tsRNAs that serve as novel diagnostic biomarkers for glioma and to investigate the mechanism by which tsRNAs regulate glioma development. These findings provide new approaches for the early diagnosis and molecular‐targeted therapy of glioma.

## 2 Materials and Methods

### 2.1 Human Blood Samples and Clinical Information of Participants

Between January 2022 and February 2023, peripheral blood samples were collected from 50 glioma patients at the First Medical Center of the Chinese PLA General Hospital before surgery. The inclusion criterion was a diagnosis of glioma based on MRI and pathological findings. The exclusion criteria were glioma recurrence, preoperative cancer treatment, fuzzy pathological diagnosis, and history of other tumors. After strict screening, 47 patients with glioma, including 25 with grades II–III and 22 with grade IV, were enrolled in this study. Of these, blood samples from eight patients with low‐grade glioma (LGG) and seven with GBM were randomly selected for small RNA sequencing. Five peripheral blood samples from healthy volunteers were used as the controls. Blood samples from the remaining 32 patients with gliomas (17 with LGG and 15 with GBM) and 32 healthy volunteers were used for RT‐qPCR analysis. Plasma samples were obtained from 5 mL of peripheral blood. Blood samples were centrifuged at 1900 × g for 10 min at 4°C, followed by 3000 × g for 15 min at 4°C. The research protocol was approved by the Medical Ethics Committee of the First Medical Center of the Chinese PLA General Hospital. Written informed consent of all participants was obtained.

### 2.2 Isolation and Identification of Plasma Exosomes

Exosomes were isolated via ultracentrifugation. Briefly, the shed microvesicles (200–1000 nm) were eliminated by centrifuging the plasma samples at 10,000 × g for 30 min. The collected supernatants were filtered through a 0.22‐μm filter followed by centrifugation at 100,000 × g for 2 h. The exosome pellets were resuspended in PBS and immediately analyzed. For transmission electron microscopy (TEM), the exosomes were placed on a copper mesh and negatively stained with 2% uranyl acetate before examination using TEM (Tecnai G2 spirit, Thermo Scientific) at 120 kV. A nanoparticle tracking analysis system was used to analyze the particle size distribution of the exosomes.

### 2.3 Small RNA Sequencing

Total RNA of plasma‐derived exosomes was extracted using TRIzol reagents (15,596,026, Invitrogen). RNA quantity was determined using a NanoDrop ND‐1000 spectrophotometer, and RNA quality was determined using a Bioanalyzer 2100 system. For each sample, 3‐OH ends were formed by dephosphorylation of total RNA. Dimethyl sulfoxide and enzymatically labeled with Cy3 were used to denature the 3‐OH‐ended RNA. The labeled RNA was hybridized onto an Arraystar Human small RNA Microarray (KC‐2208122, 8 × 15 K, Arraystar), and the array was scanned using an Agilent Scanner G2505C.

The acquired array images were analyzed by Agilent Feature Extraction (v11.0.1.1). GeneSpring GX (v12.1) was used for quantile normalization and subsequent data processing. After normalization, probe signals with Present or Marginal QC flags in at least five out of 20 samples were retained. Multiple probes from the same sRNAs (miRNAs, tsRNAs [including tRFs and tiRNAs], pre‐miRNAs, tRNAs, and snoRNAs) were combined at the RNA level. The fold‐change (FC) and *p*‐value were used to identify the differentially expressed small RNAs. Hierarchical clustering heatmaps, volcano plots, and scatter plots were created to show small RNA expression profiles among samples.

### 2.4 Reverse Transcription‐Quantitative Polymerase Chain Reaction (RT‐qPCR)

Total RNA of the exosomes derived from the plasma and cell culture supernatants for RT‐qPCR was extracted using exoRNeasy Midi Kits (77,144, Qiagen). Total RNA of glioma cells and tumor tissues was extracted using miRNeasy Mini Kits (217,004, Qiagen). miRNA 1st Strand cDNA Synthesis Kits (MR101‐01, Vazyme) or ReverAid First Strand cDNA Synthesis Kits (K1622, Thermo Scientific) were used to synthesize cDNA. RT‐qPCR was performed using GoTaq qPCR Master Mix (A5001, Promega) on an ABI 7500 system (Applied Biosystems). U6 and β‐actin were chosen as internal controls for quantifying i‐tRF‐LeuCAA and TPM4, respectively. The primers are listed in Table S1. The log(2^−(∆∆Ct)^) values of each sample were used for statistical analysis.

### 2.5 Bioinformatic Analysis

Clinical information and transcriptional data of glioma patients were downloaded from The Cancer Genome Atlas (TCGA) and Chinese Glioma Genome Atlas (CGGA). A total of 1390 glioma patients, including 697 samples from TCGA RNA sequencing data and 693 samples from CGGA RNA sequencing data, were collected and analyzed. RNA‐seq data were converted using log2 TPM transformation.

tsRNA expression data from TCGA samples were derived from MINTbase (v2.0). The expression data of differentially expressed tsRNAs obtained using small RNA sequencing were searched in the TCGA database according to FC from large to small, and the candidate tsRNAs were screened. Patients with glioma in the TCGA database were divided into two groups according to the expression level of candidate tsRNAs in glioma samples or overlapping differentially expressed genes (DEGs) in glioma cells after candidate tsRNA overexpression. Kaplan–Meier (K–M) survival curves were obtained using the Survminer R package (v3.6.3). The differential expression of the candidate tsRNAs or DEGs among the groups with different molecular diagnostic characteristics was compared using clinical information from the TCGA and CGGA databases.

The sequences of candidate tsRNAs were put into the miRDB website interface to obtain their potential target genes, and the top 50 genes with the highest correlation were selected as candidate genes for Go ontology (GO) and Kyoto Encyclopedia of Genes and Genomes (KEGG) enrichment analysis. The relationship between the top 50 potential target mRNAs and candidate tsRNAs was mapped into a network using Cytoscape (v3.7.2).

The miMap database was used to predict potential binding sites of candidate tsRNAs on potential target mRNAs. The R and limma packages were used to identify DEGs between patients with high and low expression levels of the target mRNA in TCGA and CGGA datasets, and GO and KEGG enrichment analyses of DEGs within the intersection were performed using Metascape.

### 2.6 Cell Culture and Transfection

Human glioma cell lines U251 and LN229 were cultured in Dulbecco's modified Eagle's medium (DMEM; 11,995,065, Gibco) containing 10% exosome‐free fetal bovine serum (FBS; 10,091,148, Gibco) in a 5% CO_2_ incubator at 37°C. The cell lines were reverse transfected with an i‐tRF‐LeuCAA mimic (B02003), i‐tRF‐LeuCAA inhibitor (B03001), negative control (NC) mimic (B04002) or inhibitor (B04003), small interfering RNA (siRNA) for TPM4 (A01001), or siRNA NC (siNC; A06001) obtained from GenePharma using Lipofectamine RNAiMAX (13,778,030, Invitrogen) and Opti‐MEM I Medium (31,985,070, Gibco). The mimics, inhibitors, siRNAs, and NC sequences are listed in Table S2. The specific mimic was a double‐stranded small RNA molecule designed according to the sequence of tsRNA, which could mimic the activity of endogenous tsRNA to investigate the gain‐of‐function effect. The specific inhibitor based on 21–23 nt 2′‐methoxy‐modified RNA oligonucleotide could inhibit the function of endogenous tsRNA.

### 2.7 Cell Proliferation Assay

A Cell Counting Kit‐8 (CCK‐8; CK‐04, Dojindo) was used to determine changes in cell proliferation. First, 5000 transfected glioma cells per well were seeded into 96‐well plates. Then, 10 μL of CCK‐8 solution was added at 24, 48, 72, and 96 h, followed by incubation at 5% CO_2_ and 37°C for 2 h. Subsequently, the absorbance at 450 nm of each well was measured using a Multiskan Go microplate reader (Thermo Fisher Scientific).

### 2.8 Migration and Invasion Assay

The capacity for cell migration and invasion was determined using a Transwell assay. Briefly, 1 × 10^5^ transfected glioma cells suspended in serum‐free DMEM were placed on the upper chamber precoated with (invasion) or without (migration) Matrigel (356,234, BD Bioscience) in 24‐well plates, and 10% FBS DMEM was added to the lower chamber. After 24 h, the migrated or invaded cells were fixed with paraformaldehyde, followed by staining with 0.l% crystal violet. Three randomly selected fields were used for cell counting and photography.

### 2.9 Cell Cycle and Cell Apoptosis Assay

The cell cycle change of transfected glioma cells was measured using a Cell Cycle Assay Kit (C543, Dojindo). Briefly, 2 × 10^6^ cells transfected glioma cells, rinsed once with PBS, were resuspended in 500ul Working Solution containing propidium iodide (PI) and RNase. Then the cells were incubated in the dark for 30 min at 4°C, followed by incubation at 37°C for 30 min. Finally, the cell cycle change was analyzed using BD FACSCanto II (BD Biosciences) after removing cell clusters using 100 μm filters.

The change in apoptosis level of transfected glioma cells was measured using an Annexin V Apoptosis Detection Kit (AD11, Dojindo). Briefly, the cells were digested with ethylenediaminetetraacetic acid‐free medium and resuspended in Annexin V Binding Solution. Then, 5 μL Annexin V and 5 μL PI Solution were added to the 100 μL cell suspension at a density of 1 × 10^6^ cells/mL. Then the cells were incubated in the dark for 15 min at room temperature. Finally, the apoptotic ability was analyzed using BD FACSCanto II (BD Biosciences) after adding 400 μL Annexin V Binding Solution.

### 2.10 Western Blot (WB)

Total proteins isolated from different glioma cells or glioma tissues of mice were separated in SDS‐PAGE and further transferred to PVDF membranes. Subsequently, the membranes were incubated with primary antibodies overnight at 4°C and secondary antibodies for 1 h. The primary antibodies were TPM4 (1:10000, 67,244‐1‐Ig), N‐Cadherin (1:6000, 66,219‐1‐Ig), Vimentin (1:8000, 60,330‐1‐Ig), GAPDH (1:200000, 60,004‐1‐Ig), Caspase 3/P17/P19 (1:1000, 19,677‐1‐AP), BAX (1:1000, 50,599‐2‐Ig), and α‐Tubulin (1:20000, 14,555‐1‐AP). All antibodies were purchased from Proteintech. Finally, immunoreactive bands were visualized using a chemiluminescent substrate.

### 2.11 RNA Sequencing

Total RNA of glioma cells was extracted using an miRNeasy Midi Kit (217,004, Qiagen). RNA purity and concentration were assessed using a NanoDrop 2000 spectrophotometer. RNA quantity and integrity were determined using an Agilent 2100/4200 system. RNA library construction and Illumina sequencing were performed by BerryGenomics. The generated data were subjected to strict quality filtering to obtain high‐quality clean reads for subsequent analyses. Clean reads with rRNA were aligned to the reference genome using Hisat2 software. Gene expression levels were analyzed using quantification, distribution statistics, correlation analysis, and principal component analysis. The significance of the differential expression was analyzed using edgeR. The q‐value was obtained by correcting the P‐value using multiple hypothesis testing. Genes with |log2(FC)| > 1.0 and *q* < 0.05 were considered DEGs. The overall distribution and hierarchical clustering of DEGs are shown as volcano plots and heat maps, respectively.

### 2.12 Dual‐Luciferase Reporter Assay

The mutant or wild‐type sequences of 3′‐UTR binding sites of TPM4 were subcloned into the pmirGlo vector (GenePharma). U251 cells were co‐transfected with the pmirGlo vector plus the i‐tRF‐LeuCAA or NC mimics. Finally, luciferase activity was determined.

### 2.13 Animal Experiment

The study protocol was approved by the Experimental Animal Care Committee of the First Medical Center of Chinese PLA General Hospital. Balb/c female nude mice (36; 4 weeks old) were obtained from Vital River (China) and divided into six groups (*n* = 6): mimic NC, mimic, inhibitor NC, inhibitor, mimic + siNC, and mimic + siTPM4. Syringes (10 μL) were used to stereotactically implant LN229 cells stably expressing luciferase (1 × 10^5^) into the right caudate nuclei of the mice. One week later, the mimic (agomir; B06002) and inhibitor (antagomir; B05002) of i‐tRF‐LeuCAA and NC (B04008 and B04007) and the in vivo siRNA (A09011) of TPM4 and NC (A06001) obtained from GenePharma were orthotopically injected into the tumors twice a week for 2 weeks, according to the corresponding groups. The sequences of the agomir, antagomir, siRNA, and NC are shown in Table S2. The agomir and antagomir were small RNA molecules designed according to the sequence of i‐tRF‐LeuCAA and modified with cholesterol, phosphorothioate linkage, and methoxyl. Compared with mimic and inhibitor, agomir and antagomir have higher stability in vivo. Intracranial tumor growth was observed using a bioluminescence imaging system (Berthold). Four weeks after glioma cell injection, all mice were euthanized via cervical dislocation after anesthesia with isoflurane. Brain tissues were excised for H&E staining, WB, RT‐qPCR, and Terminal deoxynucleotidyl transferase dUTP terminal labeling (TUNEL) staining analyses.

### 2.14 TUNEL Staining

Apoptotic cells in the glioma tissues of mice were detected using a TUNEL assay kit (48,513, Cell Signaling Technology) according to the instructions of the manufacturer.

### 2.15 Statistical Analyses

GraphPad Prism (v8.0) and R software were used for Statistical analyses. Data are shown as mean ± standard deviation. Unpaired two‐tailed Student's *t*‐tests were used to compare statistical differences between two groups of numerical variables, and one‐way ANOVAs with Tukey's test were used to compare statistical differences between multiple groups of numerical variables. Differences were considered significant at *p* < 0.05.

## 3 Results

### 3.1 Identification of Differentially Expressed tsRNAs in Plasma‐Derived Exosomes of Patients With Glioma

**FIGURE 1:**
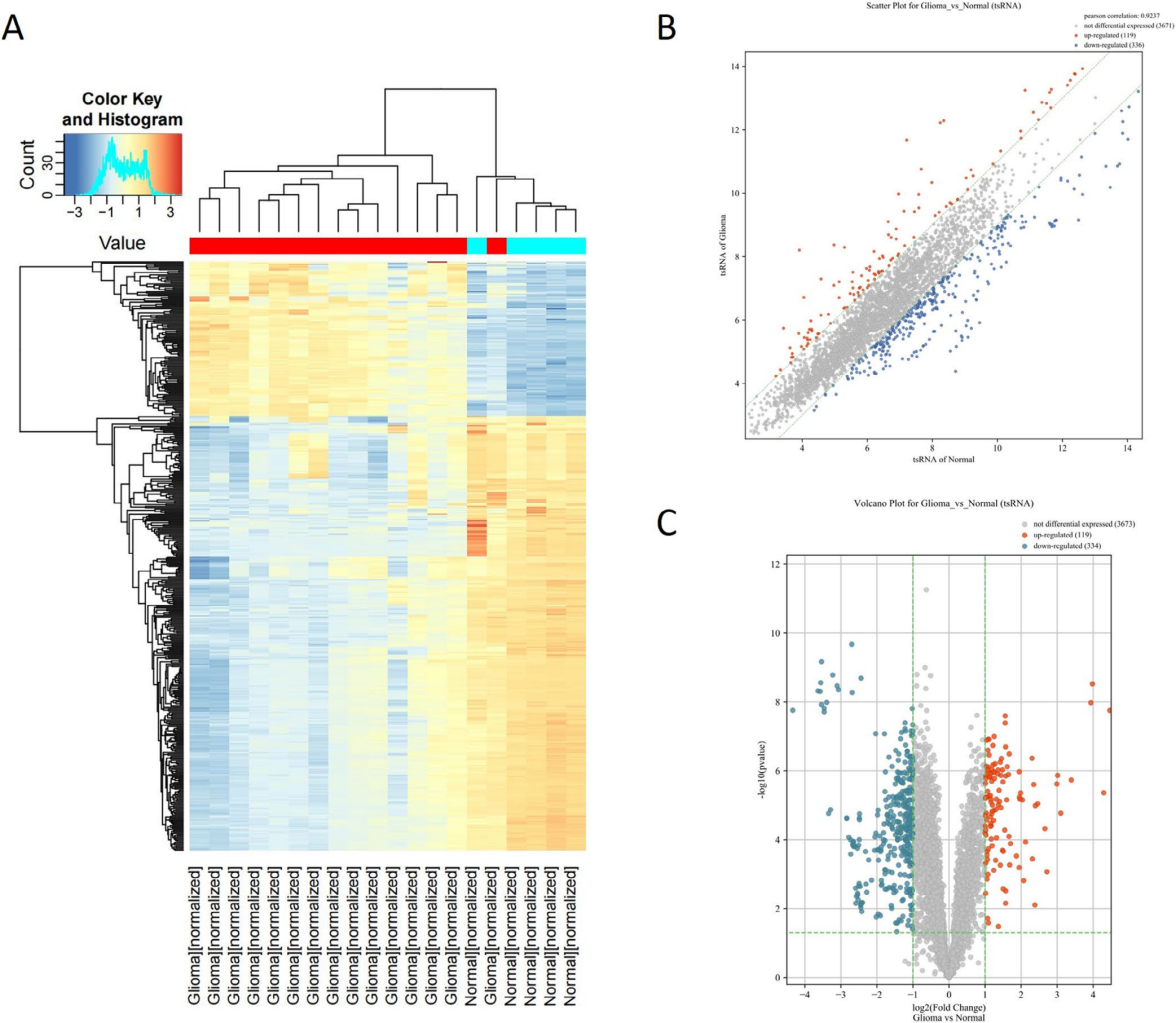
Differential expression analysis of tsRNAs in plasma exosomes from patients with glioma and healthy controls. (A) Hierarchical clustering heatmap. Each column represents one sample, and each row represents one tsRNA. (B) Scatter plot of comparison between two groups. CPM values of all tsRNAs are plotted, with *x*‐axis for normal control samples and *y*‐axis for glioma samples. tsRNAs above or below green dotted line were considered to have FC > 2. (C) Volcano plots of comparison between two groups. Values on *x*‐axis and *y*‐axis are FC converted from log2 and *p*‐values converted from −log10, respectively, among two groups. Green dotted line represents |FC| ≥ 2 and *p* < 0.05, and red/blue gradient dots represent significantly upregulated or downregulated tsRNA, respectively.

To investigate tsRNAs as potential biomarkers for glioma diagnosis, small RNA sequencing was performed for preliminary screening. First, plasma exosomes were isolated from 15 patients with glioma (seven cases of LGG and eight cases of GBM) and five healthy controls. The isolated exosomes were confirmed using TEM (Figure S1A) and nanoparticle tracking (Figure S1B). Total RNA was extracted for small RNA sequencing. As shown in the hierarchical clustering heatmap, the glioma group had a differential tsRNA expression profile compared to that of the control group (Figure 1A). Four hundred fifty‐three differentially expressed tsRNAs were identified in glioma patients, 119 upregulated with FC > 2 and 334 downregulated with FC < 0.5 (Figure 1B,C).

**FIGURE 2:**
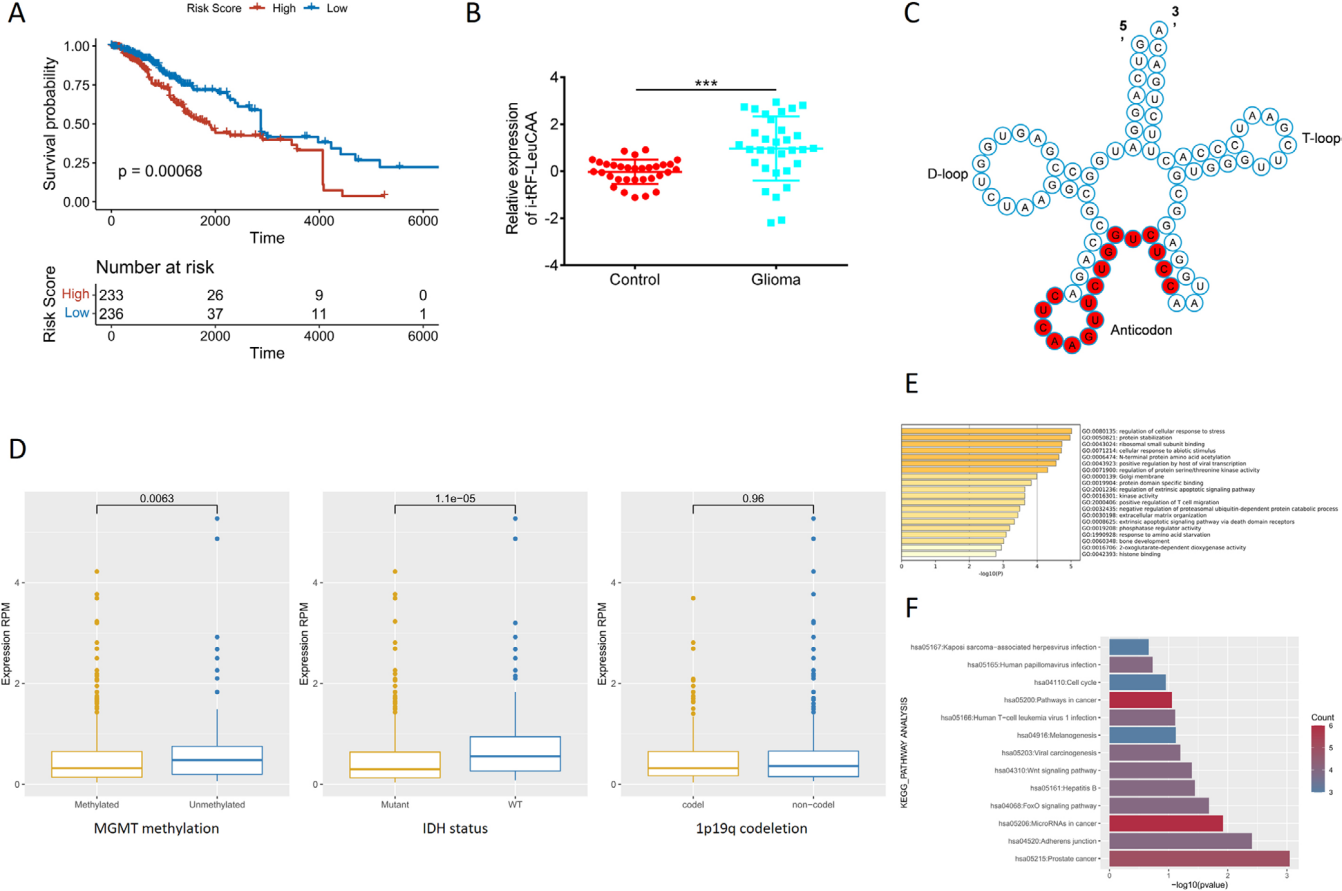
Clinical characteristics and biological function analysis of i‐tRF‐LeuCAA in gliomas. (A) K–M analysis based on the median cut‐off value of i‐tRF‐LeuCAA expression level in glioma tissue samples from TCGA‐LGG database. (B) Differential expression of i‐tRF‐LeuCAA in plasma exosomes between patients with glioma and healthy controls was validated using RT‐qPCR. ***p < 0.0001. (C) The position of i‐tRF‐LeuCAA on the secondary structure of its precursor tRNA. (D) Correlation analysis of i‐tRF‐LeuCAA with different molecular diagnostic characteristics. (E) GO enrichment analysis of potential target genes of i‐tRF‐LeuCAA. (F) KEGG pathway enrichment analysis of potential target genes of i‐tRF‐LeuCAA.

The top 10 tsRNAs upregulated in the plasma‐derived exosomes of glioma patients were selected as candidate tsRNAs (Table S3). Among them, we found the expression profile of i‐tRF‐LeuCAA in the sncRNA sequence data from the TCGA‐LGG database. Considering that LGG is the main subtype of glioma, we investigated the prognostic value of i‐tRF‐LeuCAA in glioma patients using the TCGA‐LGG database. K–M analysis demonstrated that the OS of glioma patients with low i‐tRF‐LeuCAA expression in glioma tissues was significantly higher than that of patients with high i‐tRF‐LeuCAA expression (Figure 2A). Subsequently, 32 patients with glioma and 32 healthy controls were recruited as independent training cohorts to validate the expression of i‐tRF‐LeuCAA using RT‐qPCR. The results showed that healthy controls exhibited significantly lower levels of i‐tRF‐LeuCAA in plasma exosomes than patients with glioma, demonstrating that plasma‐derived exosomal i‐tRF‐LeuCAA may be a novel biomarker for glioma diagnosis (Figure 2B).

i‐tRF‐LeuCAA was derived from the anticodon of tRNA‐LeuCAA (Figure 2C). To investigate the diagnostic value of i‐tRF‐LeuCAA further, we analyzed the correlation between i‐tRF‐LeuCAA expression in glioma tissues and different molecular diagnostic characteristics, including MGMT methylation, IDH mutations, and 1p19q codeletion. i‐tRF‐LeuCAA expression was significantly higher in patients with unmethylated MGMT than in those with methylated MGMT (Figure 2D). In addition, significantly higher expression of i‐tRF‐LeuCAA was detected in patients that were wild‐type than in those with IDH mutations (Figure 2D). Compared with the 1p/19q codeletion group, the expression of i‐tRF‐LeuCAA was slightly elevated in the non‐codeletion group (Figure 2D). Next, we predicted the potential target mRNAs of i‐tRF‐LeuCAA using the miRDB website, which is presented in an interaction network (Figure S2). Functional enrichment analyses of these mRNAs were performed to investigate the underlying mechanism of action of i‐tRF‐LeuCAAs in gliomas. GO enrichment analysis revealed that these genes mainly regulated cellular responses to stress, protein stabilization, ribosomal small subunit binding, and extracellular matrix (ECM) organization (Figure 2E). KEGG pathway analysis indicated that the enriched pathways were mainly related to cell cycle, cancer pathways, miRNAs in cancer cells, and adherens junction (Figure 2F).

### 3.2 Functional Analysis of i‐tRF‐LeuCAA in Glioma Cells

**FIGURE 3:**
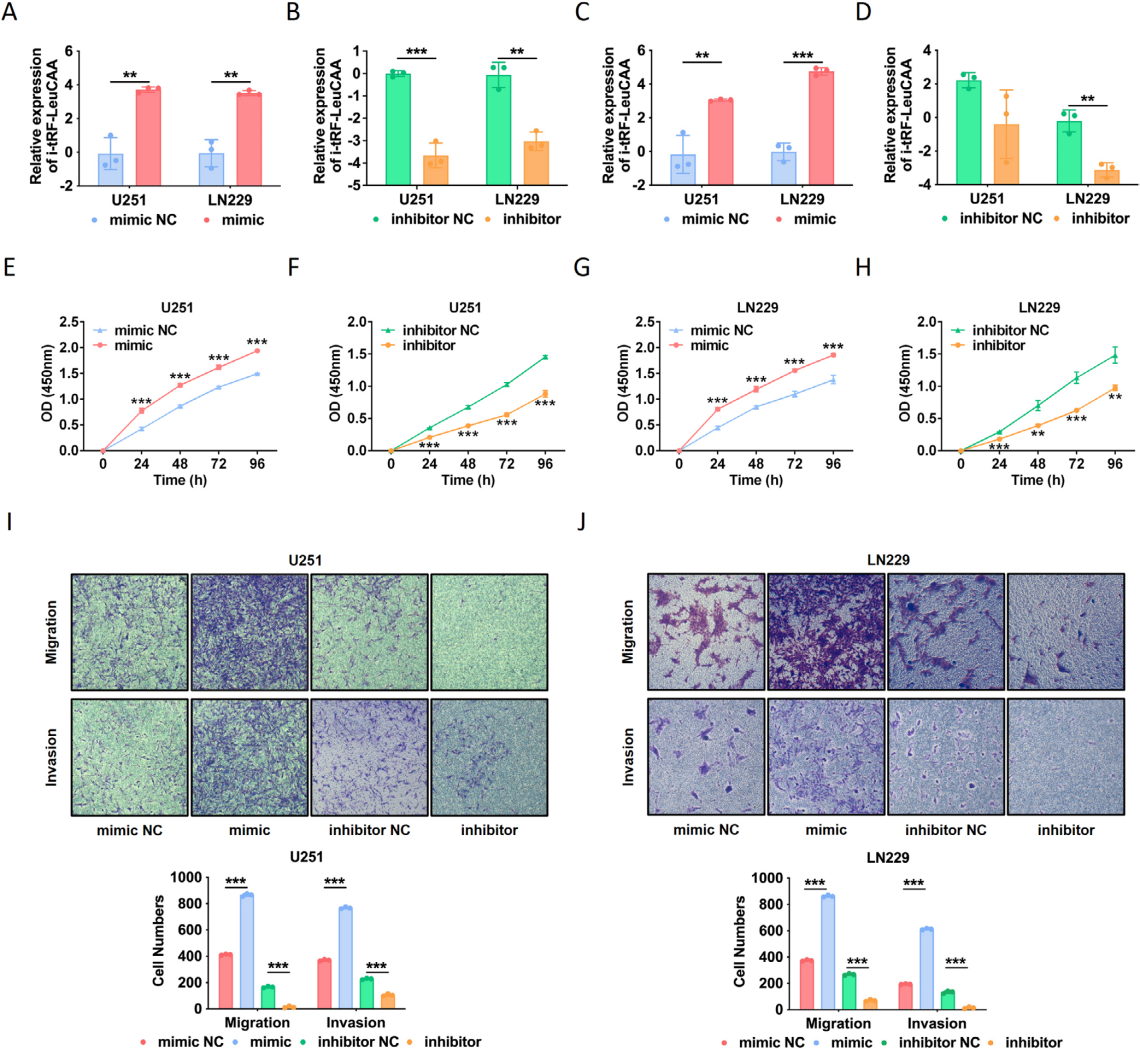
Effects of interfering with expression of i‐tRF‐LeuCAA on proliferation, migration, and invasion of glioma cells. (A) Effects of i‐tRF‐LeuCAA mimic and NC on i‐tRF‐LeuCAA expression in U251 and LN229 cells. (B) Effects of i‐tRF‐LeuCAA inhibitor and NC on i‐tRF‐LeuCAA expression in U251 and LN229 cells. (C) Effects of i‐tRF‐LeuCAA mimic and NC on i‐tRF‐LeuCAA expression in U251 and LN229 cell‐derived exosomes. (D) Effects of i‐tRF‐LeuCAA inhibitor and NC on i‐tRF‐LeuCAA expression in U251 and LN229 cell‐derived exosomes. (E–H) Effects of i‐tRF‐LeuCAA mimic, inhibitor, and NC on proliferation of U251 and LN229 cells. (I, J) Effects of i‐tRF‐LeuCAA mimic, inhibitor, and NC on migration and invasion of U251 and LN229 cells. *p < 0.05, **p < 0.01, ***p < 0.001.

To investigate the potential biological role of i‐tRF‐LeuCAA in gliomas further, specific mimics or inhibitors were used to upregulate or downregulate the expression of i‐tRF‐LeuCAA in U251 and LN229 glioma cell lines, respectively. The RT‐qPCR results indicated that the mimic significantly upregulated the expression of i‐tRF‐LeuCAA in both glioma cell lines and exosomes derived from the culture medium (Figure 3A,C). Moreover, this inhibitor downregulated i‐tRF‐LeuCAA expression (Figure 3B,D). These results indicate that increased exosomal i‐tRF‐LeuCAA was released from glioma cells. Subsequently, we evaluated the effects of i‐tRF‐LeuCAA on the proliferation, migration, invasion, and apoptosis of glioma cells in vitro. The results demonstrated that the overexpression of i‐tRF‐LeuCAA significantly increased the proliferation (Figure 3E), migration (Figure 3I), and invasion (Figure 3I) abilities of U251 cells. Similar effects were observed in LN229 cells (Figure 3G,J). Conversely, the proliferation (Figure 3F,H), migration (Figure 3I,J), and invasion (Figure 3I,J) abilities of U251 and LN229 cells treated with the i‐tRF‐LeuCAA inhibitor were significantly suppressed.

**FIGURE 4:**
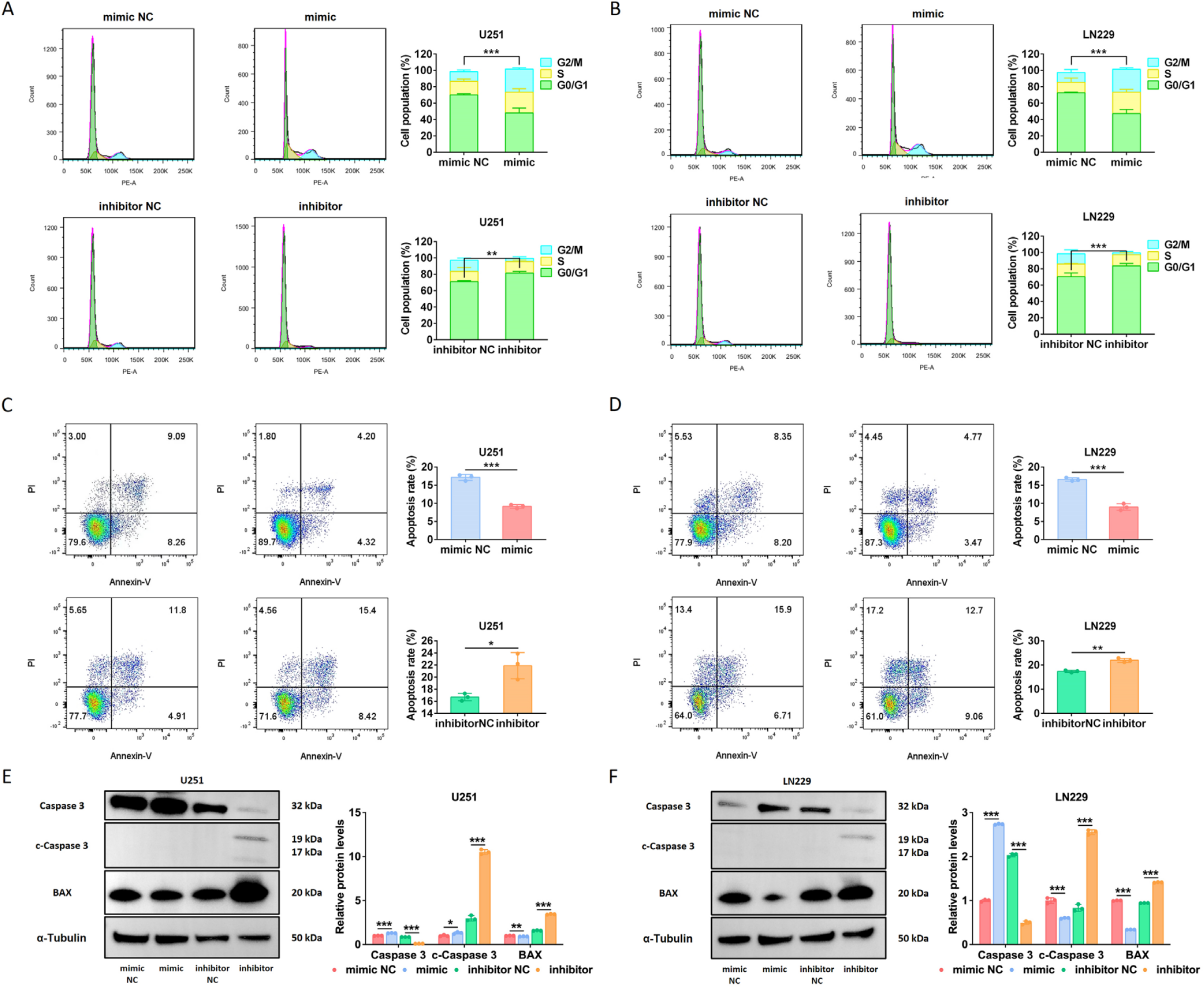
Effects of interfering with expression of i‐tRF‐LeuCAA on cell cycle and apoptosis of glioma cells. (A, B) Effects of i‐tRF‐LeuCAA mimic, inhibitor, and NC on cell cycle of U251 and LN229 cells. (C, D) Effects of i‐tRF‐LeuCAA mimic, inhibitor, and NC on apoptosis of U251 and LN229 cells. (E, F) Analysis of apoptosis‐related proteins in U251 and LN229 cells after transfection with i‐tRF‐LeuCAA mimic or inhibitor. *p < 0.05, **p < 0.01, ***p < 0.001.

The KEGG analyses indicated that i‐tRF‐LeuCAA may be related to the cell cycle (Figure 2F). The verification results demonstrated that upregulation of i‐tRF‐LeuCAA induced cell cycle arrest in the G2/M phase. Conversely, i‐tRF‐LeuCAA inhibition caused cell cycle arrest in the G0/G1 phase (Figure 4A,B). Subsequently, flow cytometry analyses revealed that the overexpression of i‐tRF‐LeuCAA significantly inhibited apoptosis in both glioma cell lines. In addition, i‐tRF‐LeuCAA inhibition increased their apoptosis ability (Figure 4C,D). The results of WB showed that i‐tRF‐LeuCAA overexpression markedly increased the expression of proprotein Caspase 3 and decreased the expression of BAX, while i‐tRF‐LeuCAA inhibition led to increased expression of cleaved Caspase 3 (c‐Caspase 3) and BAX, and decreased expression of proprotein Caspase 3 in both glioma cell lines (Figure 4E,F). These results suggest that i‐tRF‐LeuCAA influences the glioma cell phenotype.

### 3.3 Analysis of Differentially Expressed Genes in Glioma Cells After i‐tRF‐LeuCAA Overexpression

**FIGURE 5:**
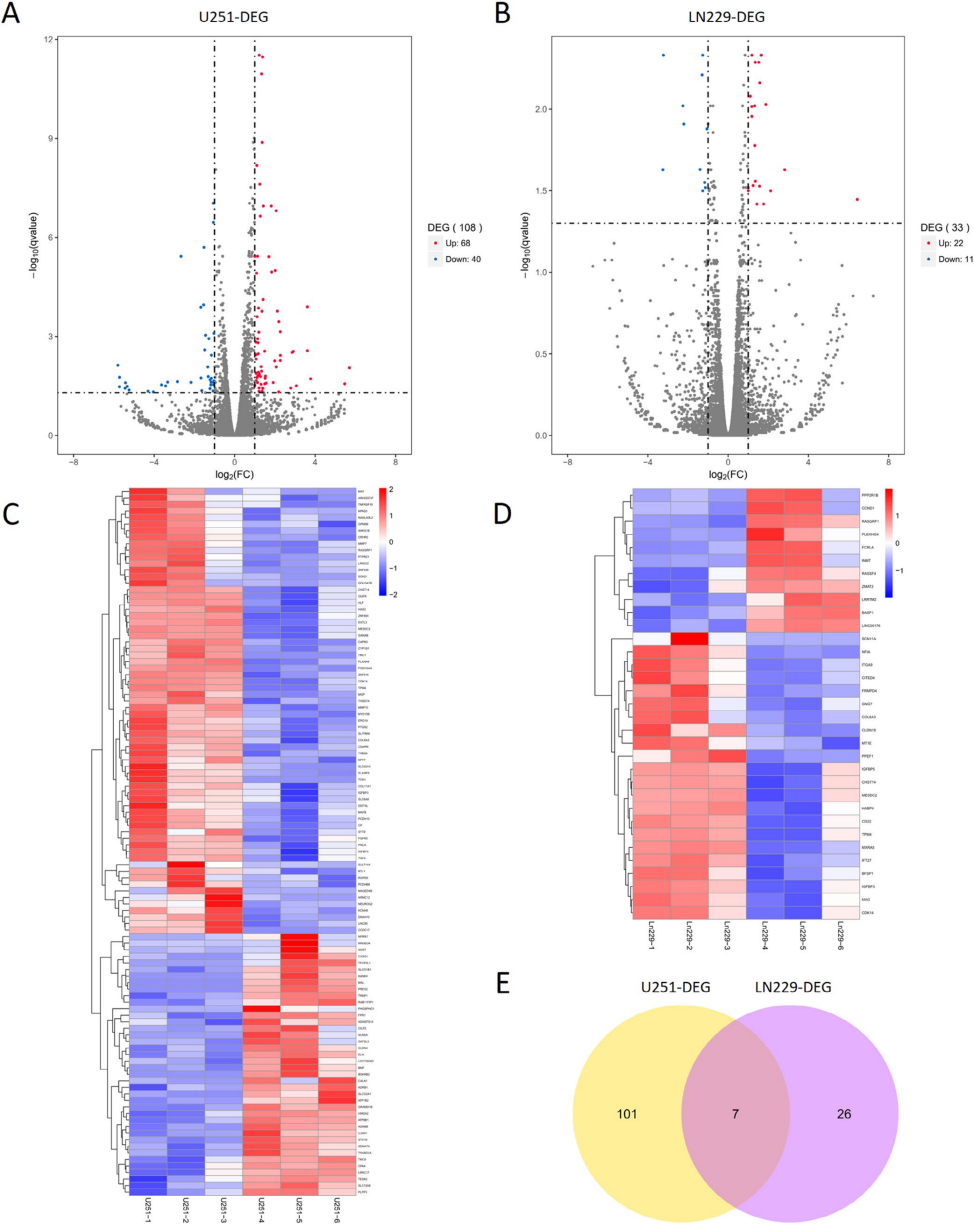
DEG analysis of glioma cells overexpressing i‐tRF‐LeuCAA. (A, B) Scatter plots of comparison between two groups in U251 and LN229 cells. *x*‐axis and *y*‐axis values are FC converted from log2 conversion and q‐value converted from −log10 among the two groups, respectively. Dotted line represented |log2(FC)| > 1.0 and *q* < 0.05. Red/blue gradient dots represent significantly upregulated and downregulated genes, respectively. (C, D) Hierarchical clustering heatmaps of DEGs in U251 and LN229 cells. (E) Venn diagrams for intersection analysis of U251‐DEGs and Ln229‐DEGs.

To identify the genes regulated by i‐tRF‐LeuCAA, we extracted total RNA from i‐tRF‐LeuCAA‐overexpressing and control group samples of U251 and LN229 cells for RNA sequencing. Compared with the control group, 108 significant DEGs were identified in U251 cells overexpressing i‐tRF‐LeuCAA, 68 upregulated and 40 downregulated (Figure 5A). In LN229 cells, 33 DEGS were identified between the i‐tRF‐LeuCAA‐overexpressing and control groups, of which 22 were upregulated and 11 were downregulated in the i‐tRF‐LeuCAA‐overexpressing group (Figure 5B). Hierarchical clustering heatmaps were constructed to visualize the DEGs between the i‐tRF‐LeuCAA‐overexpressing and control groups in the U251 and LN229 cells (Figure 5C,D).

As shown in the Venn diagram, seven DEGs were detected in both the U251 and LN229 RNA‐seq samples (Figure 5E). Seven DEGs (CDK14, MESDC2, TPM4, CHST14, IGFBP5, IGFBP3, and COL6A3) were upregulated in the i‐tRF‐LeuCAA overexpression group. Subsequently, we investigated the predictive values of these seven DEGs in patients with glioma using the TCGA glioma dataset. K–M analysis showed that patients with glioma with higher expression of TPM4, CHST14, IGFBP5, IGFBP3, or COL6A3 had significantly worse OS (Figure S3). We determined that only TPM4 and IGFBP3 contained potential binding sites for i‐tRF‐LeuCAA. Considering that TPM4 is closely associated with the malignant characteristics of gliomas, we selected TPM4 for further analysis [13]. Although there was no significant correlation between the expression levels of i‐tRF‐LeuCAA and TPM4 in the TCGA‐LGG database, glioma patients with enhanced i‐tRF‐LeuCAA expression manifested increased TPM4 expression (Figure S4).

### 3.4 Biological Function Analysis of TPM4 in Glioma

**FIGURE 6:**
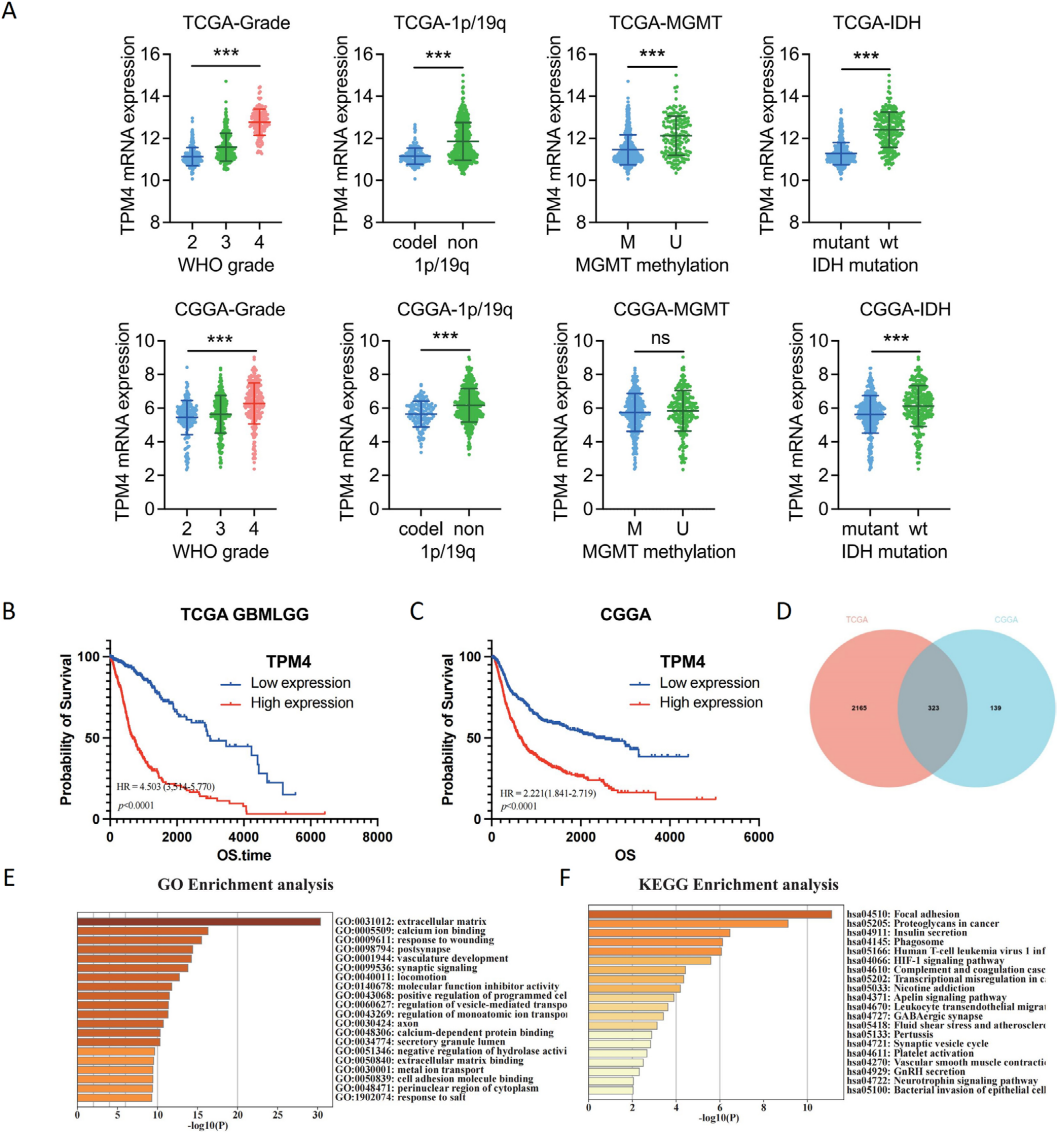
Analysis of clinical characteristics and biological functions of TPM4 in glioma. (A) TCGA and CGGA databases were used to analyze correlation between TPM4 expression and different clinical characteristics of patients with glioma. ***p < 0.001. (B, C) K–M analysis and log‐rank test based on expression level of TPM4 in glioma tissue samples in TCGA and CGGA databases, respectively. (D) Venn diagram showing overlapping DEGs related to differential expression of TPM4 in glioma tissues in TCGA and CGGA databases. (E) GO enrichment analysis of DEGs associated with differential expression of TPM4. (F) KEGG pathway enrichment analysis of DEGs associated with differential expression of TPM4.

We analyzed the associations between TPM4 expression and different molecular diagnostic characteristics of gliomas using TCGA and CGGA databases. MGMT promoter methylation, 1p/19q codeletion, and IDH mutation status are powerful predictors of improved survival times in gliomas. Patients with gliomas in these subgroups showed significantly lower TPM4 expression levels (Figure 6A). K–M analysis was used to investigate the prognostic value of TPM4 in TCGA and CGGA databases. The OS of patients with glioma with low TPM4 expression was significantly longer than that of patients with high expression in the TCGA database (Figure 6B). Similarly, a significant difference was observed in the CGGA database (Figure 6C).

To investigate the potential biological functions of TPM4, functional enrichment analyses were performed based on the DEGs between patients with glioma with high or low TPM4 expression levels. A total of 2488 and 462 DEGs were screened in TCGA and CGGA databases, respectively. A total of 323 dysregulated DEGs in both datasets were detected through intersectional analysis (Figure 6D). GO enrichment analysis revealed that these genes are mainly related to ECM (Figure 6E). The KEGG pathway analysis revealed that the enriched pathways are mainly related to focal adhesion (Figure 6F).

### 3.5 I‐tRF‐LeuCAA Influences Proliferation, Migration, Invasion, and Apoptosis of Glioma Cells by Indirectly Regulating TPM4 Expression

**FIGURE 7:**
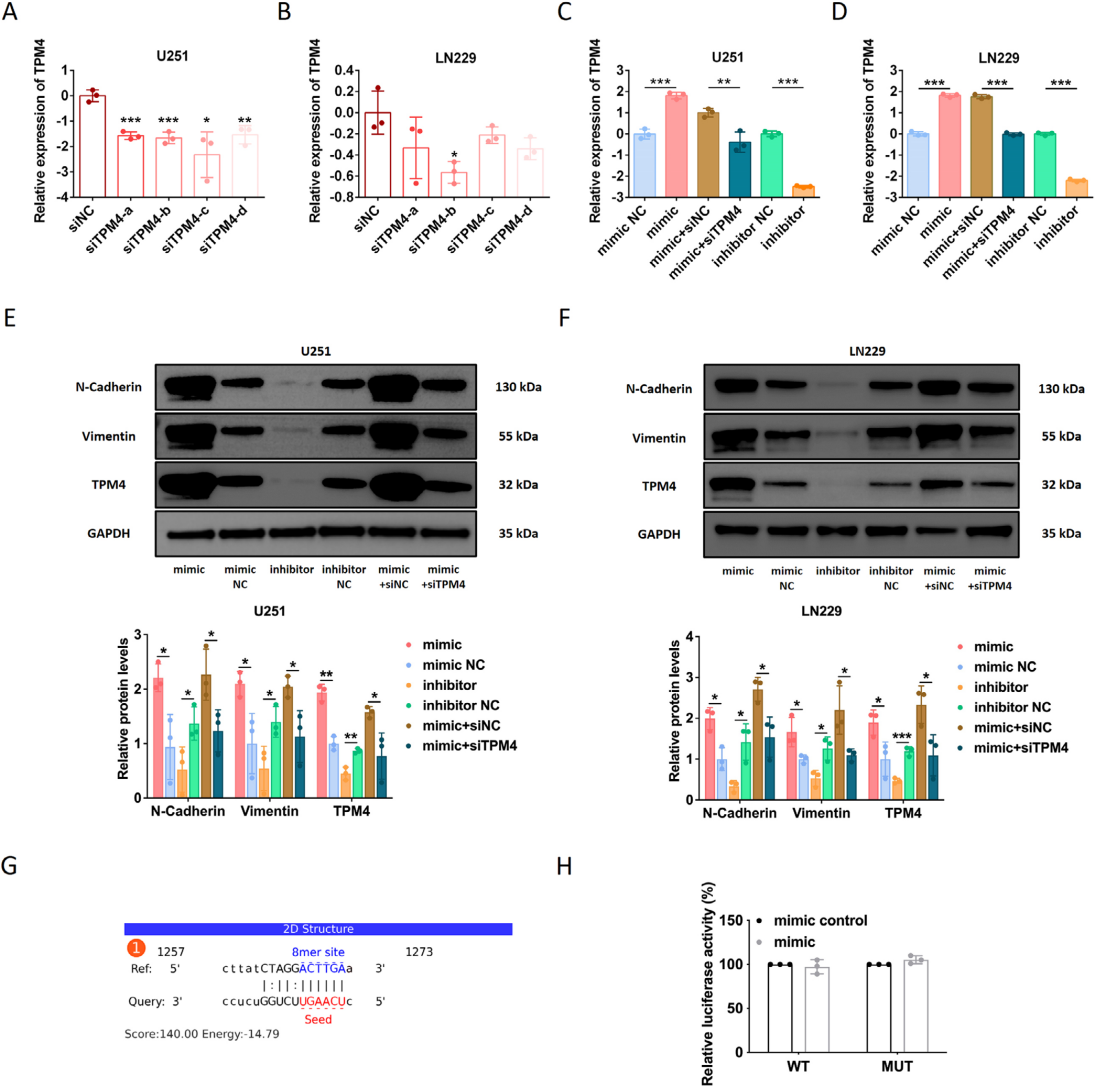
Analysis of regulatory mechanism of i‐tRF‐LeuCAA on TPM4. (A, B) Effect of siTPM4 synthesized according to four different sites on expression level of TPM4 in U251 and LN229 cells. (C, D) Analysis of TPM4 expression in U251 and LN229 cells after transfection with i‐tRF‐LeuCAA mimic, siTPM4, or i‐tRF‐LeuCAA inhibitor. (E, F) Analysis of EMT‐related markers and TPM4 protein expression in U251 and LN229 cells after transfection with i‐tRF‐LeuCAA mimic, siTPM4, or i‐tRF‐LeuCAA inhibitor. (G) Prediction of potential binding sites of i‐tRF‐LeuCAA to TPM4. (H) Binding ability of i‐tRF‐LeuCAA to TPM4 was validated using dual‐luciferase reporter assay. *p < 0.05, **p < 0.01, ***p < 0.001.

The effects of four siRNAs specifically targeting different sites of TPM4 were evaluated in the U251 and LN229 cell lines. The results of RT‐qPCR showed that siTPM4‐b inhibited TPM4 expression in both glioma cell lines (Figure 7A,B). Thereafter, we detected a change in TPM4 expression in glioma cells after knocking down i‐tRF‐LeuCAA expression. Compared to the control groups, the mRNA and protein levels of TPM4 in U251 and LN229 cells were markedly upregulated after transfection with the i‐tRF‐LeuCAA mimic but significantly downregulated after transfection with the i‐tRF‐LeuCAA inhibitor (Figure 7C–F). In addition, the effect of the i‐tRF‐LeuCAA mimic was reversed via siTPM4 (Figure 7C–F).

Bioinformatic analysis revealed that the biological function of TPM4 was closely related to the ECM (Figure 6E). Moreover, a previous study detected a strong relationship between TPM4 and epithelial–mesenchymal transition (EMT) signaling pathways in glioma through Gene Set Variation Analysis [13]. Considering that EMT is closely correlated with various cytokines and signaling pathways in the ECM, we measured changes in the protein expression of EMT‐related markers. The protein levels of N‐Cadherin and Vimentin in both U251 and LN229 cells were markedly upregulated after transfection with the i‐tRF‐LeuCAA mimic but significantly downregulated after transfection with the i‐tRF‐LeuCAA inhibitor (Figure 7E,F). However, the effect of the i‐tRF‐LeuCAA mimic was reversed via siTPM4 compared with that of siNC (Figure 7E,F).

**FIGURE 8:**
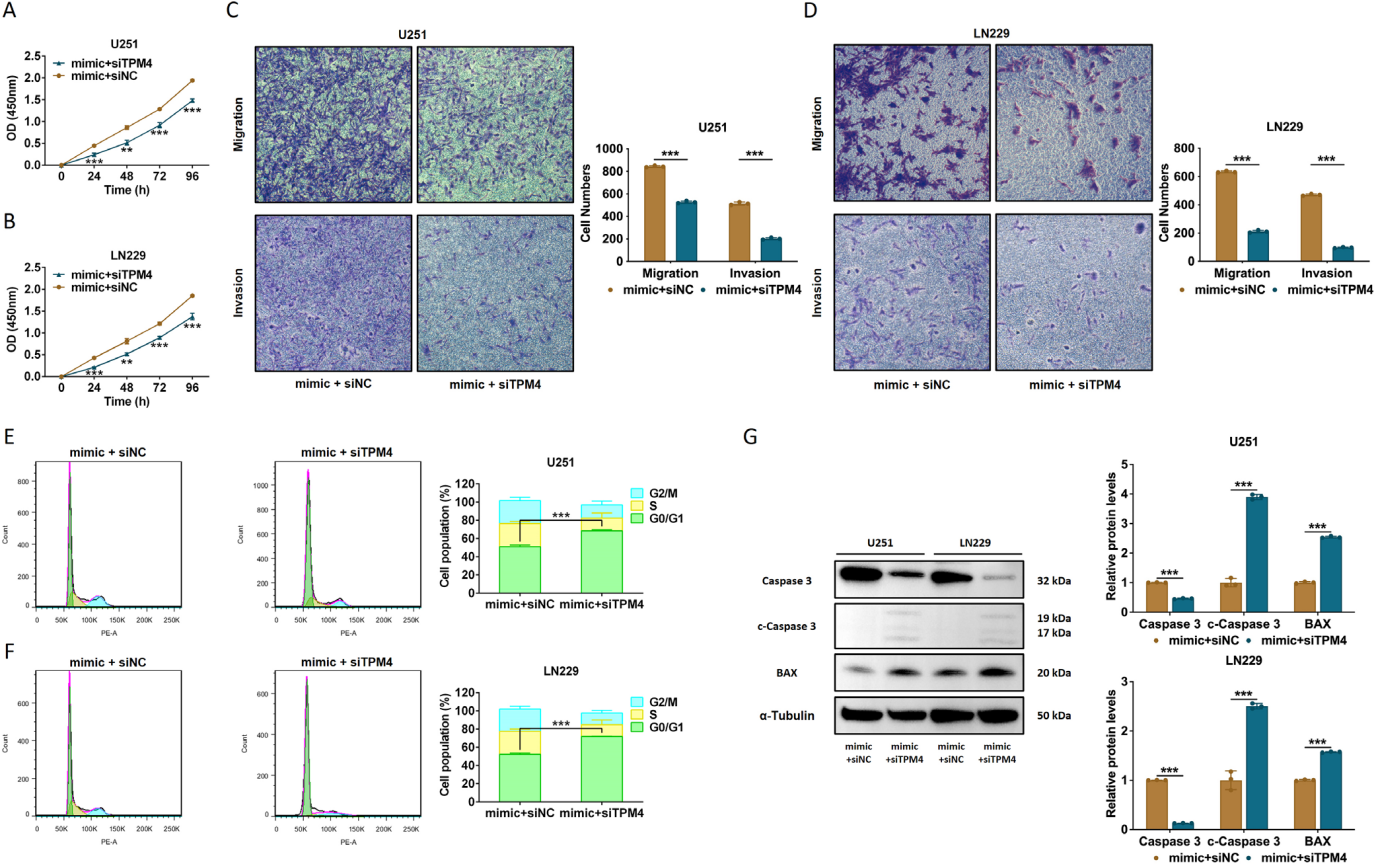
i‐tRF‐LeuCAA regulates glioma cell phenotype via TPM4. (A, B) Effects of i‐tRF‐LeuCAA mimic + siTPM4/siNC on proliferation of U251 and LN229 cells. (C, D) Effects of i‐tRF‐LeuCAA mimic + siTPM4/siNC on migration and invasion of U251 and LN229 cells. (E, F) Effects of i‐tRF‐LeuCAA mimic + siTPM4/siNC on cell cycle of U251 and LN229 cells. (G) Analysis of apoptosis‐related proteins in U251 and LN229 cells after transfection with i‐tRF‐LeuCAA mimic + siTPM4/siNC. **p < 0.01, ***p < 0.001.

TPM4 may contain binding sites for i‐tRF‐LeuCAA (Figure 7G). Dual‐luciferase reporter assays were used to validate the potential binding sites of i‐tRF‐LeuCAA and TPM4. The mimic did not influence luciferase activity in either the i‐tRF‐LeuCAA‐TPM4‐WT or i‐tRF‐LeuCAA‐TPM4‐MUT groups compared with that in the control groups (Figure 7H). These results indicated that i‐tRF‐LeuCAA indirectly regulates the expression of TPM4 in glioma cells. The effects of i‐tRF‐LeuCAA and TPM4 on glioma cell proliferation, migration, and invasion were also explored. As shown in Figure 8A,C, the promoting effect of the i‐tRF‐LeuCAA mimic on the proliferation, migration, and invasion of U251 cells was reversed via siTPM4. Similar results were also observed in LN229 cells (Figure 8B,D). In addition, siTPM4 could reverse the cell distribution reduction in G0/G1 phase in both glioma cell lines (Figure 8E,F). The results of WB showed that increased expression of proprotein Caspase 3 and decreased expression of c‐Caspase 3 and BAX induced by i‐tRF‐LeuCAA mimic could be reversed by siTPM4 (Figure 8G).

### 3.6 I‐tRF‐LeuCAA Influences Glioma Growth by Regulating TPM4 Expression

**FIGURE 9:**
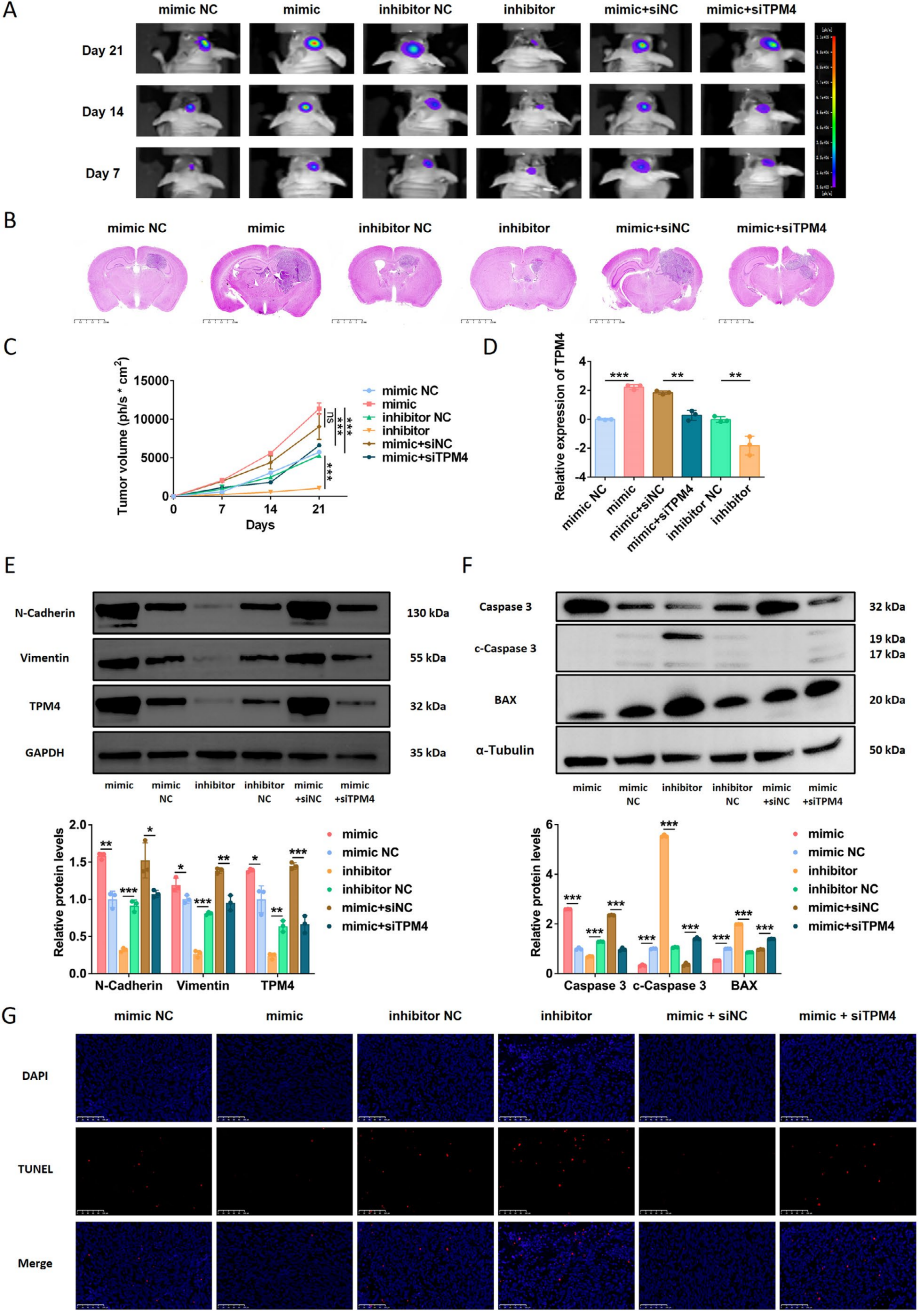
i‐tRF‐LeuCAA regulates glioma growth in vivo via TPM4. (A) Intracranial tumor size was measured using bioluminescence imaging after intracranial in situ injection of i‐tRF‐LeuCAA mimic (agomir), in vivo siTPM4, or i‐tRF‐LeuCAA inhibitor (antagomir). (B) Representative H&E staining images of brain tissue in each group. Scale bar = 2.5 mm. (C) Tumor growth curve. (D) Changes in TPM4 expression in tumor tissues of mice (data represent three independent experimental replicates). (E) Analysis of expression of EMT‐related markers and TPM4 in tumor tissues of mice (data represent three independent experimental replicates). (F) Analysis of expression of apoptosis‐related markers in tumor tissues of mice (data represent three independent experimental replicates). (G) Representative TUNEL staining images of glioma tissue in each group. DAPI, Blue; TUNEL, Red; Scale bar = 100 μm. *p < 0.05, **p < 0.01, ***p < 0.001.

Animal experiments were performed to verify the effects of i‐tRF‐LeuCAA‐regulated TPM4 on glioma growth in vivo. As shown in the bioluminescence images, H&E staining images, and tumor growth curves, i‐tRF‐LeuCAA overexpression promoted intracranial glioma growth in mice, which was reversed via siTPM4 (Figure 9A–C). Conversely, the i‐tRF‐LeuCAA inhibitor suppressed intracranial growth of glioma cells (Figure 9A–C). Consistent with the in vitro experiments, the mRNA and protein levels of TPM4 and the protein expression of EMT‐related markers in glioma tissues were significantly elevated in the i‐tRF‐LeuCAA overexpression group but remarkably downregulated in the i‐tRF‐LeuCAA inhibition group (Figure 9D,E). Additionally, the effects of the i‐tRF‐LeuCAA mimic were reversed via TPM4 inhibition in vivo (Figure 9D,E). The apoptotic proteins and apoptotic cells were also detected. Increased expression of proprotein Caspase 3, decreased expression of BAX and c‐Caspase 3, and decreased apoptosis of glioma cells could be observed in the i‐tRF‐LeuCAA overexpression group, while i‐tRF‐LeuCAA inhibition could lead to the reverse effect (Figure 9F,G). Similarly, the TPM4 inhibitor could reverse the effects of the i‐tRF‐LeuCAA mimic (Figure 9F,G). In addition, the histopathological analysis of the heart, kidney, and liver and the detection of serum alanine aminotransferase (ALT) and aspartate aminotransferase (AST) were performed to examine the potential toxic effects of orthotopically injected drugs. No obvious toxicity was observed (Figure S5).

## 4 Discussion

The poor prognosis of patients with glioma is related to an insufficient understanding of its pathogenesis and the lack of tools for timely diagnosis and sensitive monitoring of its efficacy [14]. Therefore, it is important to identify reliable biomarkers and investigate potential molecular mechanisms underlying the initiation and progression of gliomas. Qualified biomarkers can reflect the biological characteristics of tumors. Gliomas exhibit strong heterogeneity [15]. Exosomes exist in almost all body fluids and contain various bioactive molecules that can reflect the heterogeneous biological changes associated with tumors [16]. Among these bioactive molecules, ncRNAs are stably enriched in exosomes and participate in regulating tumorigenesis and glioma progression [17]. Exosomes can penetrate the blood–brain barrier, and ncRNAs can be selectively encapsulated in exosomes and secreted into the peripheral circulation, reflecting their potential as novel biomarkers [18]. ncRNAs in plasma exosomes, such as miRNAs and circRNAs, can serve as diagnostic biomarkers for glioma [19, 20, 21, 22, 23]. These findings indicate that exosomal ncRNAs have good clinical application prospects for diagnosing gliomas.

tsRNAs, usually between 18 and 40 nucleotides in length, are a new class of sncRNAs that are generated by cleaving precursor or mature tRNAs [24]. The biological characteristics of tsRNAs, such as stability enhanced by nucleotide modification, high abundance in vivo, high conservation between species, and unique expression profiles, make them important biomarkers [25, 26, 27]. Plasma‐derived tsRNAs are diagnostic biomarkers in multiple types of cancers [11, 28, 29, 30, 31], and the abundance of tsRNAs in plasma‐derived exosomes from patients with liver cancer is higher than that in healthy controls [10].

The expression levels of some tsRNAs, such as tRFdb‐3003a, tRFdb‐3003b, ts‐55, and ts‐60, were significantly decreased in glioma tissue samples and were closely related to the prognosis of glioma patients. Further investigation revealed that the expression level of tRFdb‐3003a/b was closely related to molecular characteristics, such as IDH mutation status and 1p19q codeletion, and the expression level of tRFdb‐3003a/b is significantly increased in IDH‐mutant gliomas [12]. Tu et al. detected differentially expressed tsRNAs in glioma tissues of different grades [32], and Xu et al. detected that the expression levels of three tsRNAs derived from tRNA‐Leu‐CAA are significantly lower in glioma tissues than in healthy brain tissues. Moreover, their expression levels correlate with OS, MGMT promoter methylation levels, and IDH mutation status [33]. These results reveal that tsRNAs can be served as new biomarkers for the diagnosis and prognosis of gliomas and warrant further investigation.

The microarray could simultaneously analyze a variety of small RNAs and improve the sensitivity and accuracy of detection. As a new gene sequencing technology, microarray has been applied in an accumulating body of research to investigate the potential role of sncRNAs in cancers, such as the expression pattern of small RNAs in hepatocellular carcinoma [34]. In this study, we investigated the expression profile of tsRNAs in the plasma exosomes of patients with glioma for the first time using a small RNA Microarray and detected that the expression levels of tsRNAs in the plasma‐derived exosomes of glioma patients differed from those in healthy controls. We detected that i‐tRF‐LeuCAA expression was closely related to the prognosis of glioma patients. Subsequently, we determined that the average expression level of i‐tRF‐LeuCAA in the plasma‐derived exosomes of glioma patients was significantly higher than that in healthy people by verifying more clinical samples. These results suggested that i‐tRF‐LeuCAA is a non‐invasive biomarker for the diagnosis and prognosis of glioma. To trace the origin of i‐tRF‐LeuCAA in the plasma‐derived exosomes of glioma patients, we detected the expression of i‐tRF‐LeuCAA in the exosomes of glioma cells and their culture supernatants after treatment with a mimic or inhibitor. These results indicate that the expression of i‐tRF‐LeuCAA in glioma cells and their secreted exosomes is correlated. The expression level of i‐tRF‐LeuCAA in the tumor tissues of patients with a malignant phenotype was significantly increased. The higher expression of i‐tRF‐LeuCAA in the plasma‐derived exosomes of glioma patients may have been caused by secretion from glioma cells.

Rapid proliferation and wide invasion are important characteristics of high‐grade glioma. tsRNAs regulate proliferation, the cell cycle, and apoptosis and are important in regulating tumor cell proliferation [11]. tRF‐3003a inhibits glioma cell growth in mice [12]. In this research, we detected that i‐tRF‐LeuCAA had a significant regulatory effect on glioma cell proliferation. tsRNAs also influence the metastasis of many types of tumors [11, 28, 29, 30, 31] and are important in regulating tumor cell apoptosis. tsRNAs are derived from tRNA and may have a similar function to tRNA, which could make tumor cells resistant to apoptosis [11, 28, 29, 30, 31]. Currently, there are no relevant studies on regulating the apoptosis, migration, and invasion of glioma cells. We found that interfering with the expression of i‐tRF‐LeuCAA altered the apoptosis, migration, and invasion of glioma cells.

tsRNAs can affect tumor progression by regulating RNA translation, transcription, and modification [11, 29, 30]. tRNA‐Cys‐GCA‐derived tRFdb‐3003a/b binds to the 3'‐UTR region of VAV2 and reduces the expression levels of mRNA and protein of VAV in glioma cells, regulating glioma progression [12]. Differentially expressed tsRNAs in different glioma grades may regulate NER, Hippo, and other tumor‐related signaling pathways [32]. Based on bioinformatic analysis, low tRFdb‐3012a/b expression in glioma may directly bind to the 3'‐UTR region of RBM43, and ts‐26 may directly bind to the 3'‐UTR region of HOXA13. Both these genes are associated with poor prognosis in glioma patients [33]. These results indicate that abnormally expressed tsRNAs in gliomas may regulate their progression through specific signaling pathways.

To investigate the potential target mRNA of i‐tRF‐LeuCAA, transcriptomic sequencing was performed on two glioma cell lines with different genetic backgrounds following overexpression of this tsRNA, and overlapping DEGs were identified. Subsequently, the TCGA database was used to predict the correlation between these potential target mRNAs of i‐tRF‐LeuCAA and the prognosis of patients with glioma to further narrow the focus. The results showed that TPM4, which has a potential binding site for i‐tRF‐LeuCAA, was highly expressed in the glioma tissues of patients with a malignant phenotype and poor prognosis. TPM4, a member of the tropomyosin family, promotes the malignant progression of many types of tumors [35, 36, 37, 38, 39]. As an actin‐binding protein, TPM4 is an important component of the cytoskeleton in nonmuscle cells [40]. The cytoskeleton, composed of tropomyosin‐actin complexes, is a crucial regulator of cell morphology and plays a key role in cell migration and adhesion in the pathophysiology of tumor formation [40, 41, 42]. High TPM4 expression is related to malignancy in many tumor types [35, 36, 37, 38, 39].

In this study, bioinformatic analysis revealed that high TPM4 expression was correlated with multiple glioma malignancies. An increase in pathological grade is often accompanied by a poor prognosis. The expression level of TPM4 elevated with an increase in WHO grade, and this trend was consistent in TCGA and CGGA databases. The prognosis of glioma patients with wild‐type IDH is worse than that of patients with IDH mutations [43], and the results showed that the expression level of TPM4 in patients with wild‐type IDH was significantly increased. Moreover, patients with glioma with an unmethylated MGMT promoter have extremely poor sensitivity to temozolomide chemotherapy [1], and we found that the expression level of TPM4 in this group of patients was significantly higher than that in those with MGMT promoter methylation. Higher TPM4 expression also occurred in the 1p/19q non‐codeletion subgroup of patients. These results indicate that TPM4 expression is associated with the molecular diagnostic characteristics and pathological grade of gliomas.

The results of enrichment analyses showed that the function of TPM4 was closely associated with the ECM, which plays a crucial role in tumor cell migration and invasion, suggesting that TPM4 and its related genes may interact and exploit synergy in extracellular matrix remodeling, migration, and invasion of glioma cells. TPM4 promotes the invasion, migration, and metastasis of various types of tumors [35, 44, 45]. EMT is closely related to various cytokines and signaling pathways that are crucial for tumor cell invasion and distant spread [46, 47] and is a key mechanism in glioma migration, invasion, and treatment resistance [48, 49, 50]. TPM4 is closely related to EMT and may play a vital role in promoting EMT through synergistic interactions with key molecules [13]. Combined with previous findings, we speculate that TPM4 may be involved in EMT in gliomas. Subsequently, we confirmed that interference with i‐tRF‐LeuCAA expression in glioma cells influenced the expression of TPM4 at both the mRNA and protein levels. The effects of the interaction between i‐tRF‐LeuCAA and TPM4 on the proliferation, migration, and invasion of glioma cells in vitro and in vivo were verified. These results demonstrated that TPM4 reversed the effect of i‐tRF‐LeuCAA in promoting glioma progression. During EMT, E‐cadherin is converted to N‐cadherin, and the expression levels of Vimentin and N‐cadherin increase [51, 52, 53]. The results showed that the expression levels of mesenchymal cell markers in glioma cells and intracranial glioma tissues of mice were significantly increased after interference with the expression of i‐tRF‐LeuCAA, indicating that i‐tRF‐LeuCAA promotes EMT in glioma cells by regulating TPM4 expression.

The results of the dual‐luciferase reporter assay indicated that i‐tRF‐LeuCAA did not promote the proliferation, migration, or invasion of glioma cells by directly binding to a specific site on TPM4 mRNA, suggesting that i‐tRF‐LeuCAA may indirectly regulate the expression of TPM4 in glioma cells. However, the exact underlying mechanism is complex and requires further investigation. tsRNAs play a vital role in the positive regulation of translation. For instance, ribosomal protein S28 (RPS28) mRNA contains two targets that bind to LeuCAG‐3' tsRNA, one of which is located in the coding sequence and forms a double strand with itself. Another target forms a folded secondary hairpin structure containing the initiation site of translation and is located in the non‐coding 3'‐UTR sequence [54]. After binding to RPS28 mRNA, LeuCAG‐3' tsRNAs unfold the secondary structure, which ultimately promotes translation in tumor cells [55]. In addition, the tRNAThr‐3' half binds to multimers and ribosomes and promotes synthesis of the protein by facilitating mRNA loading into the ribosomes with active translation [56]. The 5'‐tRF Gln19, 19 nucleotides in length, promotes the translation of ribosomes and poly(A) binding proteins by interacting with the Human Multisynthetase Complex [57]. The increased expression of 33‐nt‐GlyGCC 5'‐tRNA half in the tissues of papillary thyroid carcinoma binds to RBM17 and promotes the movement of RBM17 into the nucleus, and the localization of RBM17 in the nucleus improves its stability by preventing its degradation by the ubiquitin/proteasome [58]. Stabilized RBM17 induces alternative splicing of exon 16 on MAP4K4 mRNA, further promoting the migration and proliferation of papillary thyroid carcinoma cells through the MAPK signaling pathway [58]. These results provided theoretical support for further investigations of the transcriptional translation of TPM4, which i‐tRF‐LeuCAA positively regulates in glioma cells.

This work had some limitations. First, there are few public databases related to tsRNAs. Relevant information could only be retrieved from the non‐coding small RNA sequencing data of the TCGA‐LGG database but not from the TCGA‐GBM and CGGA databases. In the future, we will consistently focus on constantly updated exosome‐related public databases and attempt to discover more tsRNAs with value in diagnosing glioma. Second, the sample size should be increased in future studies. Third, tsRNAs have abundant modifications that may make them more stable, which may also be closely related to the various biological characteristics of glioma cells. However, the biological role of the i‐tRF‐LeuCAA modification in gliomas requires further investigation. Fourth, ribosome sequencing and RNA pull‐down techniques can be used to investigate the exact mechanism by which i‐tRF‐LeuCAA positively regulates TPM4 transcription and translation in subsequent studies.

**FIGURE 10:**
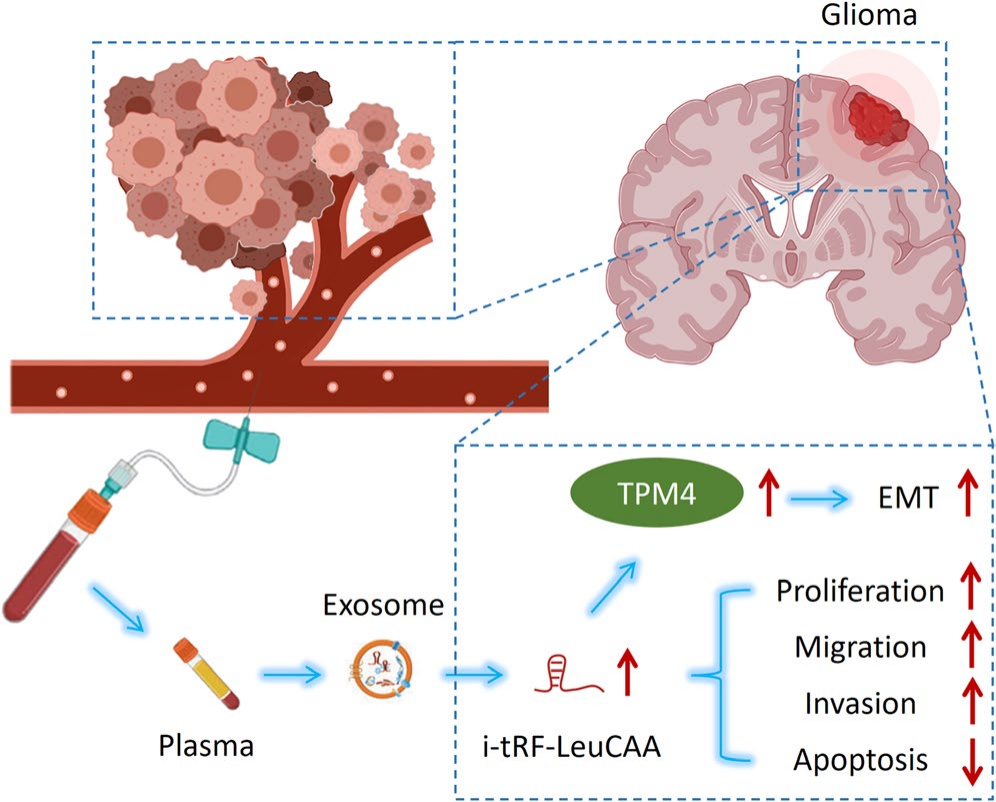
Schematic model for i‐tRF‐LeuCAA being served as a non‐invasive biomarker of glioma and promoting glioma progression by regulating TPM4 expression and influencing EMT.

Differentially expressed tsRNAs were present in the plasma‐derived exosomes of glioma patients. Among them, i‐tRF‐LeuCAA could be served as a non‐invasive biomarker in the diagnosis and prognosis of glioma and may promote the proliferation, migration, and invasion of glioma cells and inhibit their apoptosis. In addition, TPM4 is a potential target of i‐tRF‐LeuCAA, which may indirectly regulate TPM4 expression and influence EMT, promoting glioma progression (Figure 10).

## Author Contributions

H.L., W.H., and L.Z. analyzed the data and prepared the manuscript. Z.L. collected the clinical specimens and performed the experiments. J.L. and L.C. designed and supervised the work. All authors agree to be accountable for the content of the work.

## Conflicts of Interest

The authors declare that they have no competing interests. Ling Chen is an Editorial Board Member of CNS Neuroscience and Therapeutics and a co‐author of this article. To minimize bias, they were excluded from all editorial decision‐making related to the acceptance of this article for publication.

## Supporting information


**Figure S1.** Morphological characteristics of plasma‐derived exosomes in patients with gliomas. (A) TEM image showing typical characteristics of exosomes. Scale bar = 100 nm. (B) Size distribution of exosomes determined using NanoSight analysis.

**Figure S2.** Interaction networks of i‐tRF‐LeuCAA and its potential target genes.

**Figure S3.** Correlation analysis between overlapping DEGs in two glioma cell lines with i‐tRF‐LeuCAA overexpression and prognosis of patients with glioma. (A) CDK14, (B) MESDC2, (C) TPM4, (D) CHST14, (E) IGFBP5, (F) IGFBP3, (G) COL6A3.

**Figure S4.** The correlation analysis between i‐tRF‐LeuCAA expression and TPM4 expression in gliomas using TCGA‐LGG database. (A) The correlation scatter‐plots of i‐tRF‐LeuCAA and TPM4. (B) Comparison of TPM4 expression between i‐tRF‐LeuCAA high and low group.

**Figure S5.** Toxicity evaluation of indicated treatment. (A) Histopathological analysis of heart, kidney, and liver of the mice. Scale bar = 100 μm (B) Detection of serum ALT and AST of the mice.

**Table S1.** The sequences of primers.

**Table S2.** The sequences of mimics, inhibitor, agomir, antagomir, siRNA, and NC.

**Table S3.** Significantly differentially expressed tsRNA in plasma exosomes of glioma patients.

## Data Availability

The datasets used and/or analyzed during the current study are available from the corresponding author on reasonable request.

## References

[1] R. Stupp , W. P. Mason , M. J. van den Bent , et al., “Radiotherapy Plus Concomitant and Adjuvant Temozolomide for Glioblastoma,” New England Journal of Medicine 352, no. 10 (2005): 987–996, 10.1056/NEJMoa043330. 15758009

[2] Y. S. Lee , Y. Shibata , A. Malhotra , and A. Dutta , “A Novel Class of Small RNAs: tRNA‐Derived RNA Fragments (tRFs),” Genes & Development 23, no. 22 (2009): 2639–2649, 10.1101/gad.1837609. 19933153 PMC2779758

[3] M. Yu , B. Lu , J. Zhang , J. Ding , P. Liu , and Y. Lu , “tRNA‐Derived RNA Fragments in Cancer: Current Status and Future Perspectives,” Journal of Hematology & Oncology 13, no. 1 (2020): 121, 10.1186/s13045-020-00955-6. 32887641 PMC7487644

[4] S. P. Keam and G. Hutvagner , “tRNA‐Derived Fragments (tRFs): Emerging New Roles for an Ancient RNA in the Regulation of Gene Expression,” Life 5, no. 4 (2015): 1638–1651, 10.3390/life5041638. 26703738 PMC4695841

[5] M. Tkach and C. Théry , “Communication by Extracellular Vesicles: Where We Are and Where We Need to Go,” Cell 164, no. 6 (2016): 1226–1232, 10.1016/j.cell.2016.01.043. 26967288

[6] J. Saint‐Pol , F. Gosselet , S. Duban‐Deweer , G. Pottiez , and Y. Karamanos , “Targeting and Crossing the Blood‐Brain Barrier With Extracellular Vesicles,” Cells 9, no. 4 (2020): 851, 10.3390/cells9040851. 32244730 PMC7226770

[7] A. K. Rooj , M. Mineo , and J. Godlewski , “MicroRNA and Extracellular Vesicles in Glioblastoma: Small but Powerful,” Brain Tumor Pathology 33, no. 2 (2016): 77–88, 10.1007/s10014-016-0259-3. 26968172 PMC4853899

[8] P. Kumar , C. Kuscu , and A. Dutta , “Biogenesis and Function of Transfer RNA‐Related Fragments (tRFs),” Trends in Biochemical Sciences 41, no. 8 (2016): 679–689, 10.1016/j.tibs.2016.05.004. 27263052 PMC5173347

[9] K. Li , Y. Lin , Y. Luo , et al., “A Signature of Saliva‐Derived Exosomal Small RNAs as Predicting Biomarker for Esophageal Carcinoma: A Multicenter Prospective Study,” Molecular Cancer 21, no. 1 (2022): 21, 10.1186/s12943-022-01499-8. 35042519 PMC8764835

[10] L. Zhu , J. Li , Y. Gong , et al., “Exosomal tRNA‐Derived Small RNA as a Promising Biomarker for Cancer Diagnosis,” Molecular Cancer 18, no. 1 (2019): 74, 10.1186/s12943-019-1000-8. 30940133 PMC6444574

[11] Y. Zhang , X. Gu , Y. Li , Y. Huang , and S. Ju , “Multiple Regulatory Roles of the Transfer RNA‐Derived Small RNAs in Cancers,” Genes & Diseases 11, no. 2 (2024): 597–613, 10.1016/j.gendis.2023.02.053. 37692525 PMC10491922

[12] J. Ren , X. Wu , F. F. Shang , et al., “The tRNA‐Cys‐GCA Derived tsRNAs Suppress Tumor Progression of Gliomas via Regulating VAV2,” Disease Markers 2022 (2022): 8708312, 10.1155/2022/8708312. 36426134 PMC9681550

[13] J. Wang , Y. Yang , and B. Du , “Clinical Characterization and Prognostic Value of TPM4 and Its Correlation With Epithelial‐Mesenchymal Transition in Glioma,” Brain Sciences 12, no. 9 (2022): 1120, 10.3390/brainsci12091120. 36138856 PMC9497136

[14] C. Alifieris and D. T. Trafalis , “Glioblastoma Multiforme: Pathogenesis and Treatment,” Pharmacology & Therapeutics 152 (2015): 63–82, 10.1016/j.pharmthera.2015.05.005. 25944528

[15] D. F. Quail and J. A. Joyce , “The Microenvironmental Landscape of Brain Tumors,” Cancer Cell 31, no. 3 (2017): 326–341, 10.1016/j.ccell.2017.02.009. 28292436 PMC5424263

[16] G. van Niel , G. D'Angelo , and G. Raposo , “Shedding Light on the Cell Biology of Extracellular Vesicles,” Nature Reviews. Molecular Cell Biology 19, no. 4 (2018): 213–228, 10.1038/nrm.2017.125. 29339798

[17] Y. Xie , W. Dang , S. Zhang , et al., “The Role of Exosomal Noncoding RNAs in Cancer,” Molecular Cancer 18, no. 1 (2019): 37, 10.1186/s12943-019-0984-4. 30849983 PMC6408816

[18] T. R. Cech and J. A. Steitz , “The Noncoding RNA Revolution‐Trashing Old Rules to Forge New Ones,” Cell 157, no. 1 (2014): 77–94, 10.1016/j.cell.2014.03.008. 24679528

[19] J. Sun , Z. Sun , I. Gareev , et al., “Exosomal miR‐2276‐5p in Plasma Is a Potential Diagnostic and Prognostic Biomarker in Glioma,” Frontiers in Cell and Development Biology 9 (2021): 671202, 10.3389/fcell.2021.671202. PMC8204016 34141710

[20] Z. Bao , N. Zhang , W. Niu , et al., “Exosomal miR‐155‐5p Derived From Glioma Stem‐Like Cells Promotes Mesenchymal Transition via Targeting ACOT12,” Cell Death & Disease 13, no. 8 (2022): 725, 10.1038/s41419-022-05097-w. 35986010 PMC9391432

[21] Z. Liu , Z. Yang , and L. He , “Effect of miR‐29a‐3p in Exosomes on Glioma Cells by Regulating the PI3K/AKT/HIF‐1α Pathway,” Molecular Medicine Reports 27, no. 3 (2023): 72, 10.3892/mmr.2023.12959. 36799154 PMC9942261

[22] X. H. Zhang , Y. C. Song , F. Qiu , Z. C. Wang , N. Li , and F. B. Zhao , “Hypoxic Glioma Cell‐Secreted Exosomal circ101491 Promotes the Progression of Glioma by Regulating miR‐125b‐5p/EDN1,” Brain Research Bulletin 195 (2023): 55–65, 10.1016/j.brainresbull.2023.02.006. 36796652

[23] S. Zhang , N. Guan , X. Mao , J. Cui , X. Sui , and W. Guo , “Exosomal circRNA_104948 Enhances the Progression of Glioma by Regulating miR‐29b‐3p and DNMT3B/MTSS1 Signaling,” Journal of Environmental Pathology, Toxicology and Oncology 41, no. 2 (2022): 47–59, 10.1615/JEnvironPatholToxicolOncol.2021039775. 35695651

[24] L. Zhu , X. Liu , W. Pu , and Y. Peng , “tRNA‐Derived Small Non‐Coding RNAs in Human Disease,” Cancer Letters 419 (2018): 1–7, 10.1016/j.canlet.2018.01.015. 29337107

[25] F. Tuorto , R. Liebers , T. Musch , et al., “RNA Cytosine Methylation by Dnmt2 and NSun2 Promotes tRNA Stability and Protein Synthesis,” Nature Structural & Molecular Biology 19, no. 9 (2012): 900–905, 10.1038/nsmb.2357. 22885326

[26] Y. Zhang , Y. Zhang , J. Shi , et al., “Identification and Characterization of an Ancient Class of Small RNAs Enriched in Serum Associating With Active Infection,” Journal of Molecular Cell Biology 6, no. 2 (2014): 172–174, 10.1093/jmcb/mjt052. 24380870

[27] P. M. Godoy , N. R. Bhakta , A. J. Barczak , et al., “Large Differences in Small RNA Composition Between Human Biofluids,” Cell Reports 25, no. 5 (2018): 1346–1358, 10.1016/j.celrep.2018.10.014. 30380423 PMC6261476

[28] Y. Pekarsky , V. Balatti , and C. M. Croce , “tRNA‐Derived Fragments (tRFs) in Cancer,” Journal of Cell Communication and Signaling 17, no. 1 (2023): 47–54, 10.1007/s12079-022-00690-2. 36036848 PMC10030754

[29] A. Di Fazio and M. Gullerova , “An Old Friend With a New Face: tRNA‐Derived Small RNAs With Big Regulatory Potential in Cancer Biology,” British Journal of Cancer 128, no. 9 (2023): 1625–1635, 10.1038/s41416-023-02191-4. 36759729 PMC10133234

[30] N. Yang , R. Li , R. Liu , et al., “The Emerging Function and Promise of tRNA‐Derived Small RNAs in Cancer,” Journal of Cancer 15, no. 6 (2024): 1642–1656, 10.7150/jca.89219. 38370372 PMC10869971

[31] S. Lee , J. Kim , P. N. Valdmanis , and H. K. Kim , “Emerging Roles of tRNA‐Derived Small RNAs in Cancer Biology,” Experimental & Molecular Medicine 55, no. 7 (2023): 1293–1304, 10.1038/s12276-023-01038-5. 37430089 PMC10393972

[32] M. Tu , Z. Zuo , C. Chen , X. Zhang , S. Wang , and Y. Sun , “Transfer RNA‐Derived Small RNAs (tsRNAs) Sequencing Revealed a Differential Expression Landscape of tsRNAs Between Glioblastoma and Low‐Grade Glioma,” Gene 855 (2023): 147114, 10.1016/j.gene.2022.147114. 36526122

[33] B. Xu , J. Liang , H. Zou , J. Wang , Y. Xiong , and J. Pei , “Identification of Novel tRNA‐Leu‐CAA‐Derived tsRNAs for the Diagnosis and Prognosis of Diffuse Gliomas,” Cancer Management and Research 14 (2022): 2609–2623, 10.2147/cmar.S367020. 36072386 PMC9441585

[34] W. Yang , Y. Liu , J. Wang , et al., “Optimizing of a Suitable Protocol for Isolating Tissue‐Derived Extracellular Vesicles and Profiling Small RNA Patterns in Hepatocellular Carcinoma,” Liver International 44, no. 10 (2024): 2672–2686, 10.1111/liv.16011. 39037259

[35] X. Zhao , M. Jiang , and Z. Wang , “TPM4 Promotes Cell Migration by Modulating F‐Actin Formation in Lung Cancer,” Oncotargets and Therapy 12 (2019): 4055–4063, 10.2147/ott.S198542. 31239699 PMC6554522

[36] L. Li , T. Ye , Q. Zhang , X. Li , L. Ma , and J. Yan , “The Expression and Clinical Significance of TPM4 in Hepatocellular Carcinoma,” International Journal of Medical Sciences 18, no. 1 (2021): 169–175, 10.7150/ijms.49906. 33390785 PMC7738955

[37] X. Zhou , X. Zhu , J. Yao , X. Wang , and N. Wang , “Comprehensive Analysis of Clinical Prognosis and Molecular Immune Characterization of Tropomyosin 4 in Pancreatic Cancer,” Investigational New Drugs 39, no. 6 (2021): 1469–1483, 10.1007/s10637-021-01128-z. 33983530

[38] Y. Yan , J. Li , M. Ye , Z. Li , and S. Li , “Tropomyosin Is Potential Markers for the Diagnosis and Prognosis of Bladder Cancer,” Disease Markers 2022 (2022): 6936262, 10.1155/2022/6936262. 35734544 PMC9208974

[39] C. C. N. Wang , C. Y. Li , J. H. Cai , et al., “Identification of Prognostic Candidate Genes in Breast Cancer by Integrated Bioinformatic Analysis,” Journal of Clinical Medicine 8, no. 8 (2019): 1160, 10.3390/jcm8081160. 31382519 PMC6723760

[40] J. J. Lin , R. D. Eppinga , K. S. Warren , and K. R. McCrae , “Human Tropomyosin Isoforms in the Regulation of Cytoskeleton Functions,” Advances in Experimental Medicine and Biology 644 (2008): 201–222, 10.1007/978-0-387-85766-4_16. 19209824

[41] J. E. Clayton , L. W. Pollard , G. G. Murray , and M. Lord , “Myosin Motor Isoforms Direct Specification of Actomyosin Function by Tropomyosins,” Cytoskeleton (Hoboken) 72, no. 3 (2015): 131–145, 10.1002/cm.21213. 25712463 PMC4414888

[42] M. Janco , T. T. Bonello , A. Byun , et al., “The Impact of Tropomyosins on Actin Filament Assembly Is Isoform Specific,” BioArchitecture 6, no. 4 (2016): 61–75, 10.1080/19490992.2016.1201619. 27420374 PMC6085118

[43] K. S. Choi , S. H. Choi , and B. Jeong , “Prediction of IDH Genotype in Gliomas With Dynamic Susceptibility Contrast Perfusion MR Imaging Using an Explainable Recurrent Neural Network,” Neuro‐Oncology 21, no. 9 (2019): 1197–1209, 10.1093/neuonc/noz095. 31127834 PMC7594560

[44] M. Kabbage , M. Trimeche , H. Ben Nasr , et al., “Tropomyosin‐4 Correlates With Higher SBR Grades and Tubular Differentiation in Infiltrating Ductal Breast Carcinomas: An Immunohistochemical and Proteomics‐Based Study,” Tumour Biology 34, no. 6 (2013): 3593–3602, 10.1007/s13277-013-0939-0. 23812729

[45] Z. G. Sheng and M. H. Chen , “TPM4 Aggravates the Malignant Progression of Hepatocellular Carcinoma Through Negatively Regulating SUSD2,” European Review for Medical and Pharmacological Sciences 24, no. 9 (2020): 4756–4765, 10.26355/eurrev_202005_21164. 32432739

[46] T. Tsuji , S. Ibaragi , and G. F. Hu , “Epithelial‐Mesenchymal Transition and Cell Cooperativity in Metastasis,” Cancer Research 69, no. 18 (2009): 7135–7139, 10.1158/0008-5472.Can-09-1618. 19738043 PMC2760965

[47] J. P. Thiery , “Epithelial‐Mesenchymal Transitions in Tumour Progression,” Nature Reviews. Cancer 2, no. 6 (2002): 442–454, 10.1038/nrc822. 12189386

[48] C. Li , H. Zheng , W. Hou , et al., “RETRACTED ARTICLE: Long Non‐Coding RNA linc00645 Promotes TGF‐β‐Induced Epithelial–Mesenchymal Transition by Regulating miR‐205‐3p‐ZEB1 Axis in Glioma,” Cell Death & Disease 10, no. 10 (2019): 717, 10.1038/s41419-019-1948-8. 31558707 PMC6763487

[49] H. Li , J. Li , L. Chen , et al., “HERC3‐Mediated SMAD7 Ubiquitination Degradation Promotes Autophagy‐Induced EMT and Chemoresistance in Glioblastoma,” Clinical Cancer Research 25, no. 12 (2019): 3602–3616, 10.1158/1078-0432.Ccr-18-3791. 30862693

[50] H. Li , J. Li , G. Zhang , et al., “HMGB1‐Induced p62 Overexpression Promotes Snail‐Mediated Epithelial‐Mesenchymal Transition in Glioblastoma Cells via the Degradation of GSK‐3β,” Theranostics 9, no. 7 (2019): 1909–1922, 10.7150/thno.30578. 31037147 PMC6485286

[51] J. I. Casal and R. A. Bartolomé , “Beyond N‐Cadherin, Relevance of Cadherins 5, 6 and 17 in Cancer Progression and Metastasis,” International Journal of Molecular Sciences 20, no. 13 (2019): 3373, 10.3390/ijms20133373. 31324051 PMC6651558

[52] M. O. Nowicki , J. L. Hayes , E. A. Chiocca , and S. E. Lawler , “Proteomic Analysis Implicates Vimentin in Glioblastoma Cell Migration,” Cancers 11, no. 4 (2019): 466, 10.3390/cancers11040466. 30987208 PMC6521049

[53] I. Appolloni , M. Barilari , S. Caviglia , E. Gambini , E. Reisoli , and P. Malatesta , “A Cadherin Switch Underlies Malignancy in High‐Grade Gliomas,” Oncogene 34, no. 15 (2015): 1991–2002, 10.1038/onc.2014.122. 24858041

[54] H. K. Kim , J. Xu , K. Chu , et al., “A tRNA‐Derived Small RNA Regulates Ribosomal Protein S28 Protein Levels After Translation Initiation in Humans and Mice,” Cell Reports 29, no. 12 (2019): 3816–3824, 10.1016/j.celrep.2019.11.062. 31851915 PMC7451100

[55] H. K. Kim , G. Fuchs , S. Wang , et al., “A Transfer‐RNA‐Derived Small RNA Regulates Ribosome Biogenesis,” Nature 552, no. 7683 (2017): 57–62, 10.1038/nature25005. 29186115 PMC6066594

[56] R. Fricker , R. Brogli , H. Luidalepp , et al., “A tRNA Half Modulates Translation as Stress Response in Trypanosoma Brucei,” Nature Communications 10, no. 1 (2019): 118, 10.1038/s41467-018-07949-6. PMC6328589 30631057

[57] S. P. Keam , A. Sobala , S. Ten Have , and G. Hutvagner , “tRNA‐Derived RNA Fragments Associate With Human Multisynthetase Complex (MSC) and Modulate Ribosomal Protein Translation,” Journal of Proteome Research 16, no. 2 (2017): 413–420, 10.1021/acs.jproteome.6b00267. 27936807

[58] L. Han , H. Lai , Y. Yang , et al., “A 5'‐tRNA Halve, tiRNA‐Gly Promotes Cell Proliferation and Migration via Binding to RBM17 and Inducing Alternative Splicing in Papillary Thyroid Cancer,” Journal of Experimental & Clinical Cancer Research 40, no. 1 (2021): 222, 10.1186/s13046-021-02024-3. 34225773 PMC8256553

