# Supplementary figures and images for: Plasma‐Derived Exosomal i‐tRF‐LeuCAA as Biomarker for Glioma Diagnosis and Promoter of Epithelial‐Mesenchymal Transition via TPM4 Regulation
Source: CNS Neurosci Ther. 2025 Apr 9;31(4):e70356. doi: 10.1111/cns.70356 (PMC11979793; doi:10.1111/cns.70356)

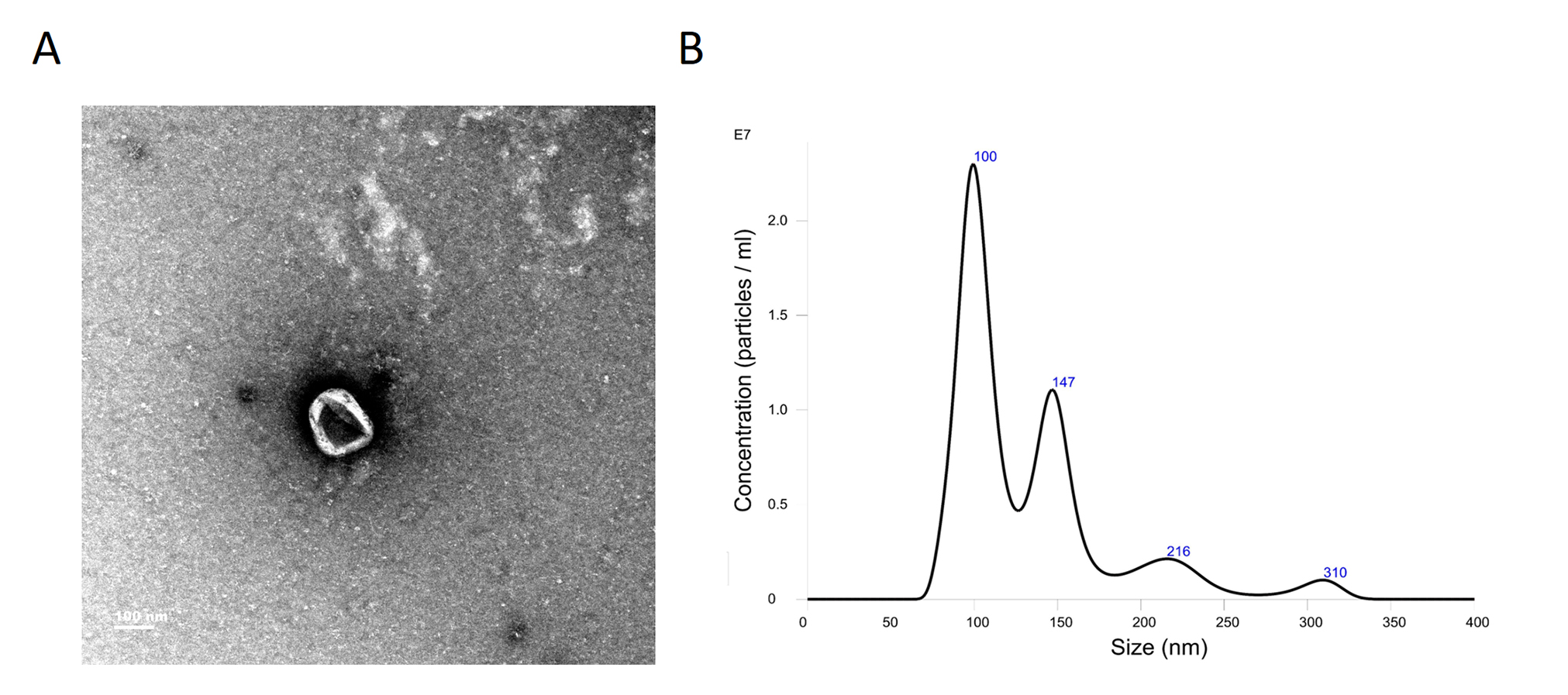

Supplement: Supplementary file 1 — Figure S1. Morphological characteristics of plasma‐derived exosomes in patients with gliomas. (A) TEM image showing typical characteristics of exosomes. Scale bar = 100 nm. (B) Size distribution of exosomes determined using NanoSight analysis. [file CNS-31-e70356-s007.jpg]

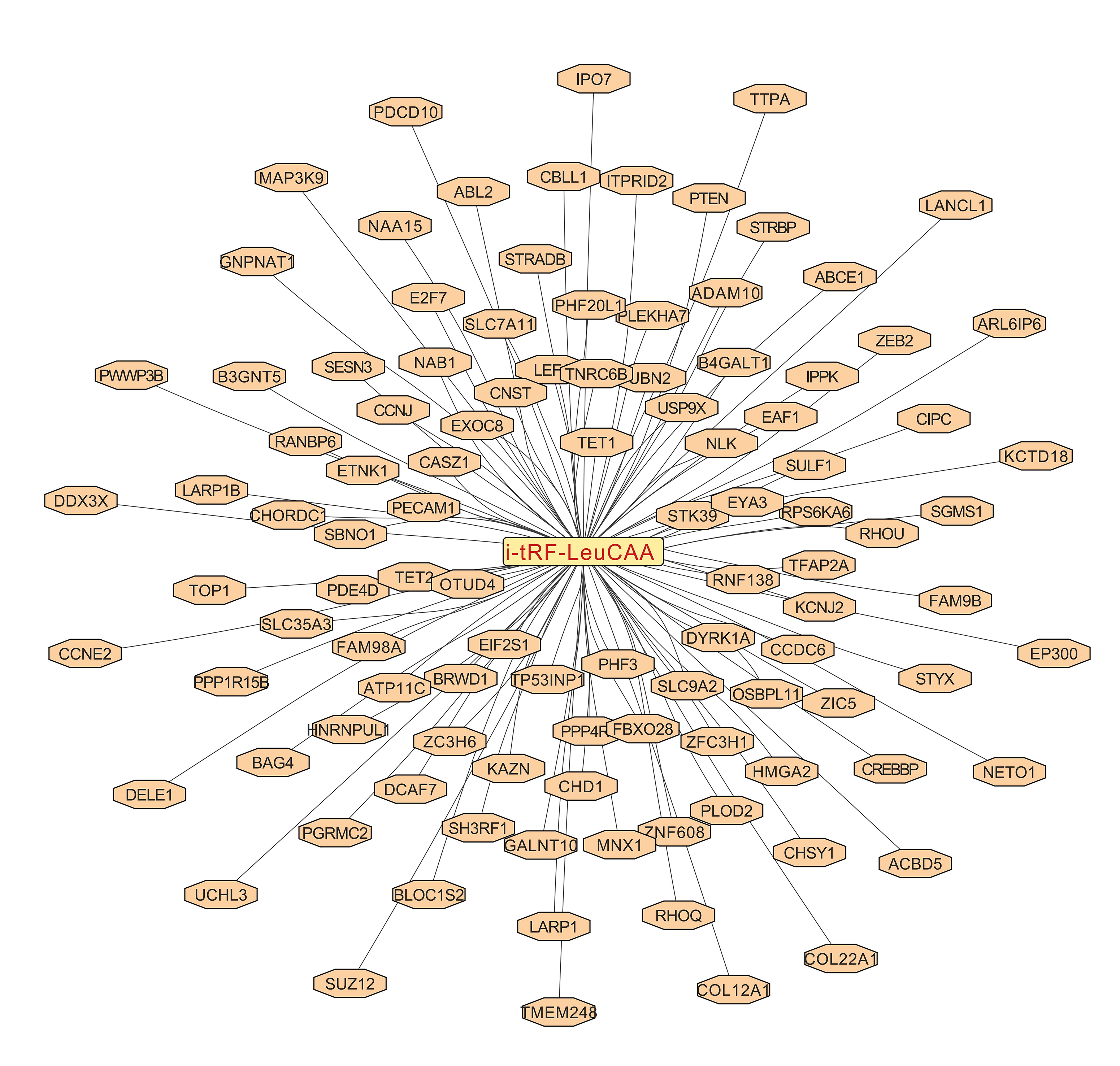

Supplement: Supplementary file 2 — Figure S2. Interaction networks of i‐tRF‐LeuCAA and its potential target genes. [file CNS-31-e70356-s004.jpg]

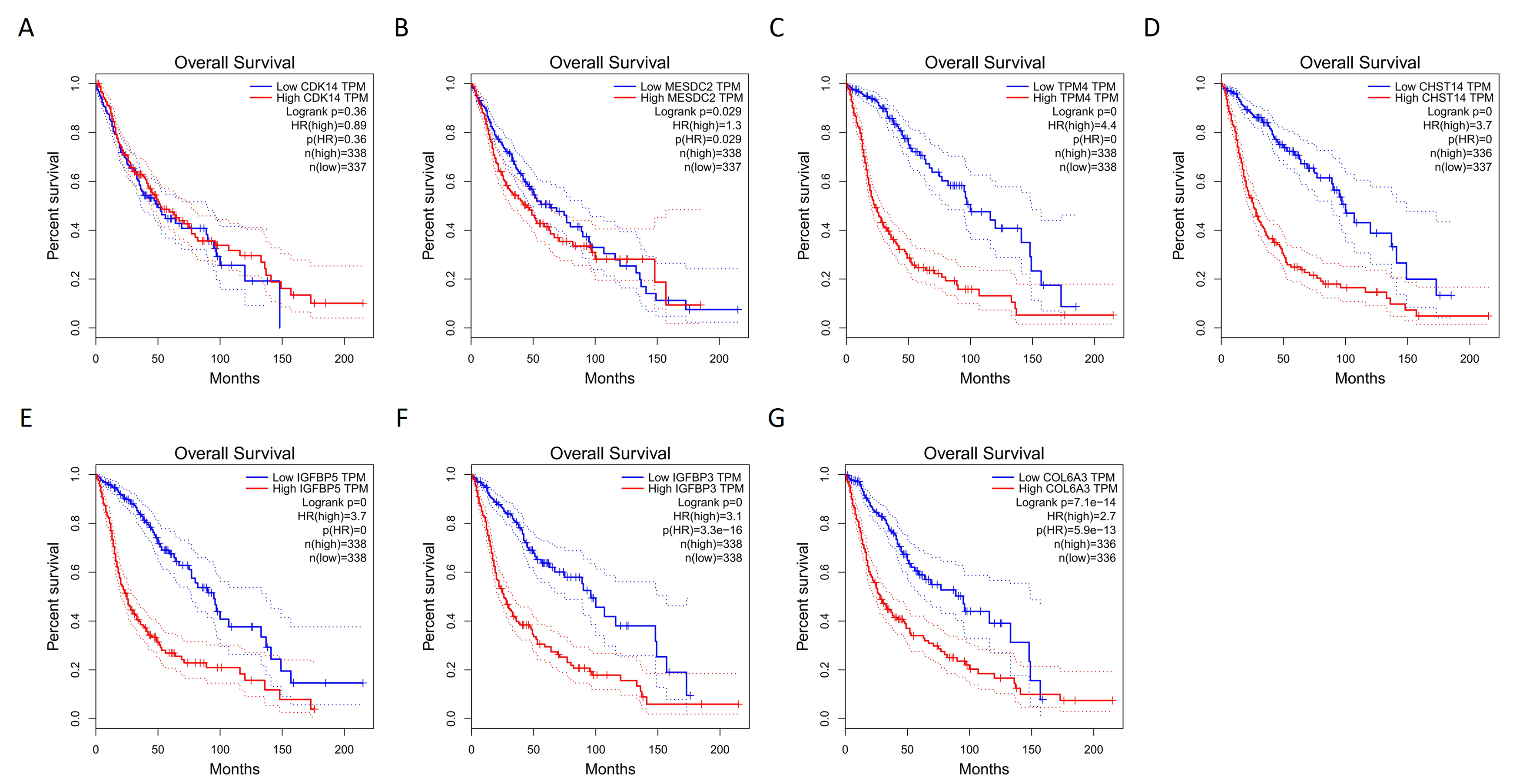

Supplement: Supplementary file 3 — Figure S3. Correlation analysis between overlapping DEGs in two glioma cell lines with i‐tRF‐LeuCAA overexpression and prognosis of patients with glioma. (A) CDK14, (B) MESDC2, (C) TPM4, (D) CHST14, (E) IGFBP5, (F) IGFBP3, (G) COL6A3. [file CNS-31-e70356-s008.jpg]

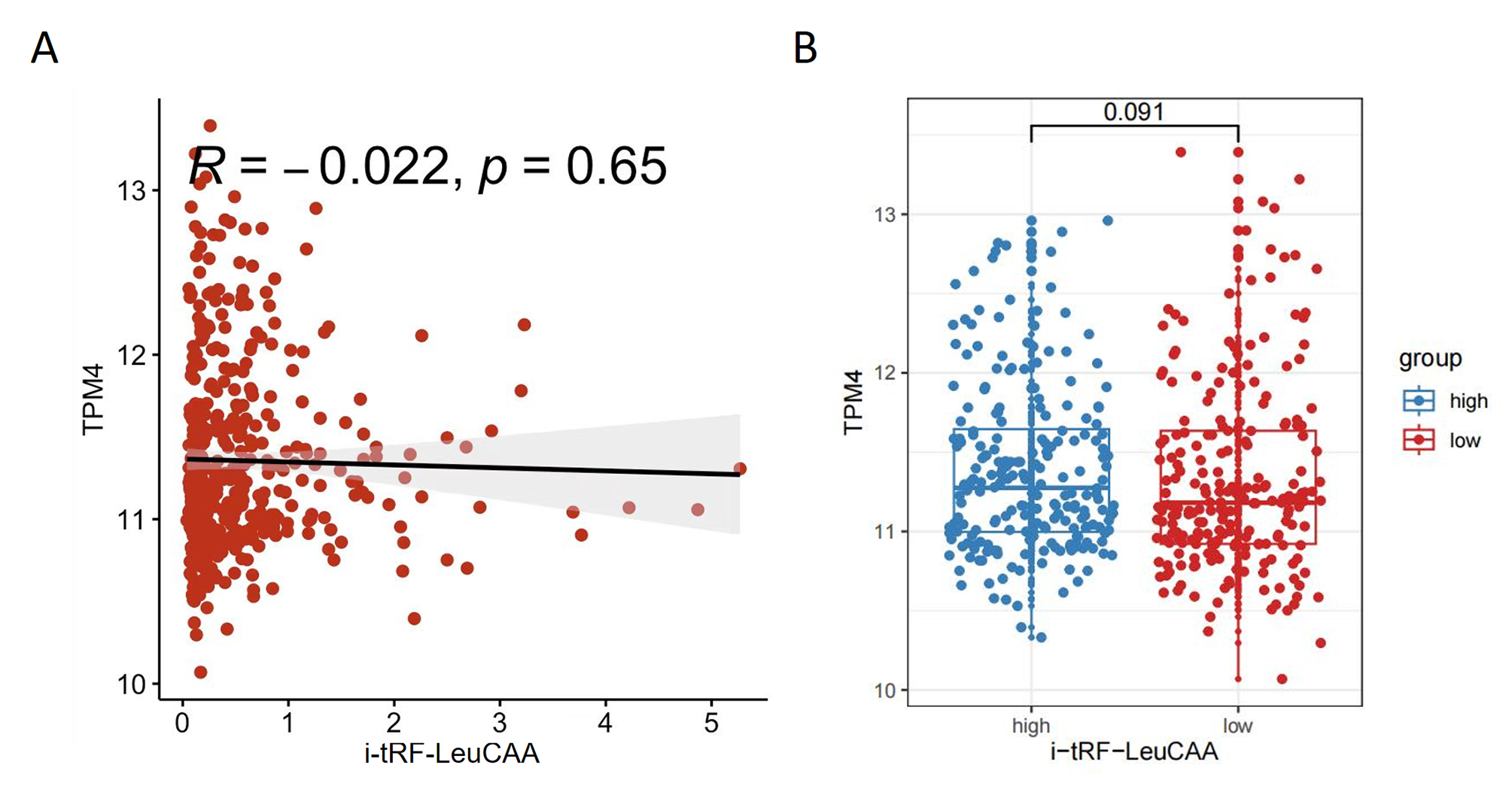

Supplement: Supplementary file 4 — Figure S4. The correlation analysis between i‐tRF‐LeuCAA expression and TPM4 expression in gliomas using TCGA‐LGG database. (A) The correlation scatter‐plots of i‐tRF‐LeuCAA and TPM4. (B) Comparison of TPM4 expression between i‐tRF‐LeuCAA high and low group. [file CNS-31-e70356-s005.jpg]

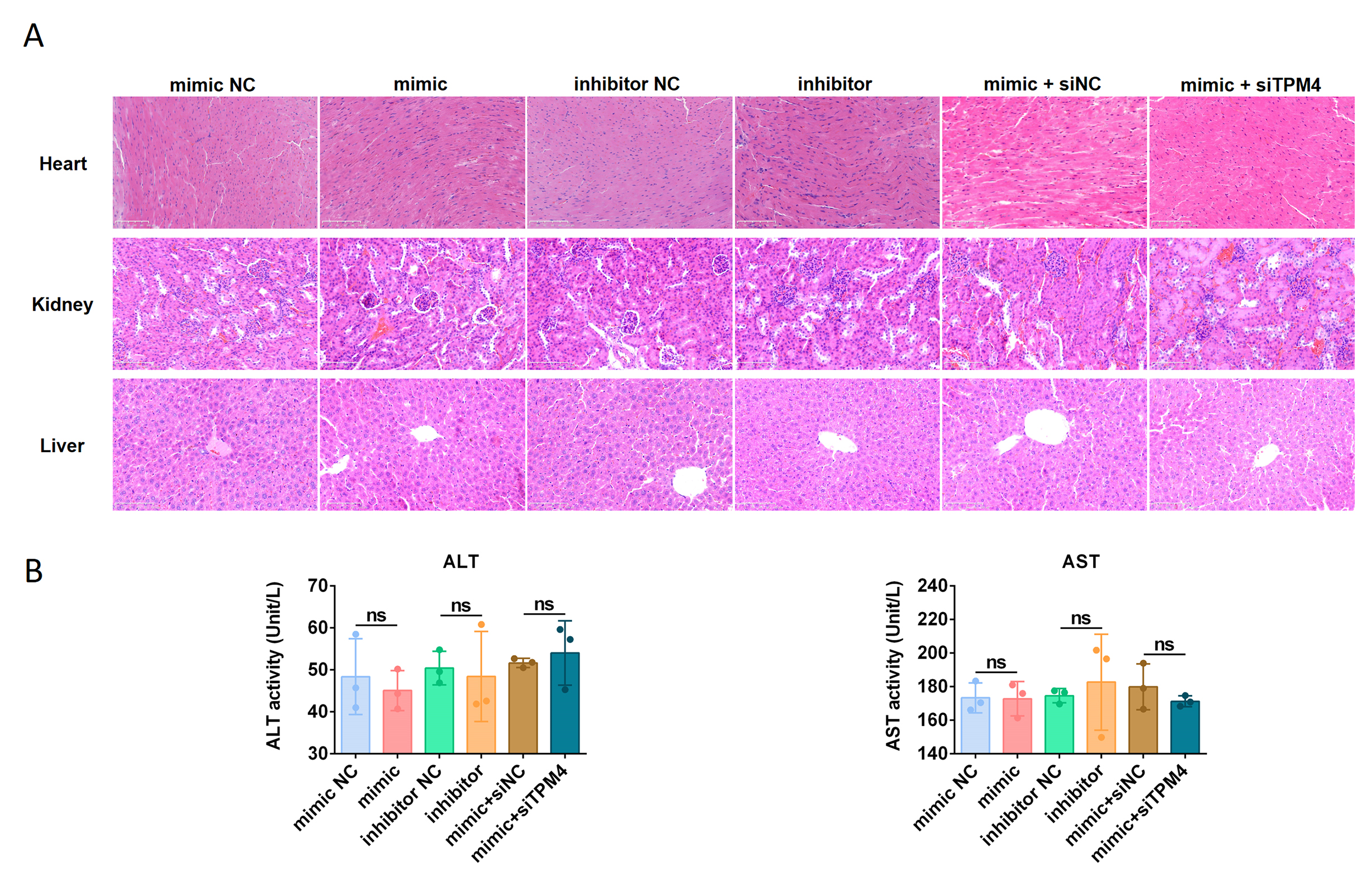

Supplement: Supplementary file 5 — Figure S5. Toxicity evaluation of indicated treatment. (A) Histopathological analysis of heart, kidney, and liver of the mice. Scale bar = 100 μm (B) Detection of serum ALT and AST of the mice. [file CNS-31-e70356-s006.jpg]
