# Supplementary material for: Plasma‐Derived Exosomal i‐tRF‐LeuCAA as Biomarker for Glioma Diagnosis and Promoter of Epithelial‐Mesenchymal Transition via TPM4 Regulation
Source: CNS Neurosci Ther. 2025 Apr 9;31(4):e70356. doi: 10.1111/cns.70356 (PMC11979793; doi:10.1111/cns.70356)
Supplement: Supplementary file 6 — Table S1. The sequences of primers. [file CNS-31-e70356-s001.docx]

**Table S1**. The sequences of primers

| **Name** | **Sequence (5’-3’)** |
| --- | --- |
| RT primer of i-tRF-LeuCAA | GTCGTATCCAGTGCAGGGTCCGAGGTATTCGCACTGGATACGACGGAGAC |
| Forward primer of i-tRF-LeuCAA | CGCGCGCTCAAGTTCTG |
| Reverse primer of i-tRF-LeuCAA | AGTGCAGGGTCCGAGGTATT |
| Forward primer of U6 | CTCGCTTCGGCAGCACA |
| Reverse primer of U6 | AACGCTTCACGAATTTGCGT |
| Forward primer of TPM4 | GTCTGACAAACTGAAAGAGGCTG |
| Reverse primer of TPM4 | CCAGTGTCTGATGTAAGCCCA |
| Forward primer of GAPDH | CACCCACTCCTCCACCTTTG |
| Reverse primer of GAPDH | CCACCACCCTGTTGCTGTAG |
