# Supplementary material for: Plasma‐Derived Exosomal i‐tRF‐LeuCAA as Biomarker for Glioma Diagnosis and Promoter of Epithelial‐Mesenchymal Transition via TPM4 Regulation
Source: CNS Neurosci Ther. 2025 Apr 9;31(4):e70356. doi: 10.1111/cns.70356 (PMC11979793; doi:10.1111/cns.70356)
Supplement: Supplementary file 7 — Table S2. The sequences of mimics, inhibitor, agomir, antagomir, siRNA, and NC. [file CNS-31-e70356-s002.docx]

**Table S2**. The sequences of mimics, inhibitor, agomir, antagomir, siRNA, and NC

| **Name** | **Sequence (5’-3’)** |
| --- | --- |
| i-tRF-LeuCAA mimic | F：CUCAAGUUCUGGUCUCC  R：AGACCAGAACUUGAGUU |
| i-tRF-LeuCAA inhibitor | GGAGACCAGAACUUGAG |
| mimic NC | F：UUCUCCGAACGUGUCACGUTT  R：ACGUGACACGUUCGGAGAATT |
| inhibitor NC | CAGUACUUUUGUGUAGUACAA |
| i-tRF-LeuCAA agomir | F：CUCAAGUUCUGGUCUCC  R：AGACCAGAACUUGAGUU |
| i-tRF-LeuCAA antagomir | GGAGACCAGAACUUGAG |
| agomir NC | F：UUCUCCGAACGUGUCACGUTT  R：ACGUGACACGUUCGGAGAATT |
| antagomir NC | CAGUACUUUUGUGUAGUACAA |
| siTPM4-Homo-219 | F：GCACCUCCAGAAGAAACUATT  R：UAGUUUCUUCUGGAGGUGCTT |
| siTPM4-Homo-537 | F：GGCCAAGCACAUUGCGGAATT  R：UUCCGCAAUGUGCUUGGCCTT |
| siTPM4-Homo-738 | F：GGAGGACAAAUAUGAAGAATT  R：UUCUUCAUAUUUGUCCUCCTT |
| siTPM4-Homo-634 | F：GCGGAGGUGUCUGAACUAATT  R：UUAGUUCAGACACCUCCGCTT |
| NC | F：UUCUCCGAACGUGUCACGUTT  R：ACGUGACACGUUCGGAGAATT |
