# Supplementary material for: Plasma‐Derived Exosomal i‐tRF‐LeuCAA as Biomarker for Glioma Diagnosis and Promoter of Epithelial‐Mesenchymal Transition via TPM4 Regulation
Source: CNS Neurosci Ther. 2025 Apr 9;31(4):e70356. doi: 10.1111/cns.70356 (PMC11979793; doi:10.1111/cns.70356)
Supplement: Supplementary file 8 — Table S3. Significantly differentially expressed tsRNA in plasma exosomes of glioma patients. [file CNS-31-e70356-s003.docx]

**Table S3**. Significantly differentially expressed tsRNA in plasma exosomes of glioma patients

| **tsRNA name** | **tsRNA-ID** | **FC** | **Regulation** | **P value** | **tsRNA**  **type** | **tsRNA**  **length** |
| --- | --- | --- | --- | --- | --- | --- |
| 5'tRF-ValCAC | tRF5-50- ValCAC-1 | 22.05373279 | up | 1.76883E-08 | 5'tRF | 50 |
| tRF-1-  ValAAC | tRF-1- ValAAC-1-1 | 19.57914219 | up | 4.3449E-06 | tRF-1 | 17 |
| i-tRF-ValCAC | i-tRF-33:55- Val-CAC-1 | 15.76652324 | up | 3.00511E-09 | i-tRF | 23 |
| i-tRF-ValCAC | i-tRF-31:54- Val-CAC-1 | 15.2959408 | up | 1.05818E-08 | i-tRF | 24 |
| 3'tiRNA- TyrGTA | 3'tiRNA-41- TyrGTA-10 | 10.51790209 | up | 1.84872E-06 | 3'tiRNA | 41 |
| 5'Leader- ProCGG | 5'Leader- ProCGG-1-2 | 8.576582183 | up | 1.69751E-05 | 5'Leader | 20 |
| 3'tiRNA- mtGlyTCC | mt-3'tiRNA-40-GlyTCC | 8.086884277 | up | 1.3613E-06 | 3'tiRNA | 40 |
| 3'tRF-TyrGTA | tRF3b- TyrGTA-10 | 7.955032217 | up | 2.40669E-06 | 3'tRF | 22 |
| i-tRF-ValTAC | i-tRF-24:39- Val-TAC-1 | 6.576620791 | up | 0.000850308 | i-tRF | 16 |
| i-tRF-  LeuCAA | i-tRF-32:e14- Leu-CAA-1 | 6.333639497 | up | 4.77715E-05 | i-tRF | 17 |
